# CCND3 is indispensable for the maintenance of B-cell acute lymphoblastic leukemia

**DOI:** 10.1038/s41389-021-00377-0

**Published:** 2022-01-10

**Authors:** Franz Ketzer, Hend Abdelrasoul, Mona Vogel, Ralf Marienfeld, Markus Müschen, Hassan Jumaa, Thomas Wirth, Alexey Ushmorov

**Affiliations:** 1 Institute of Physiological Chemistry, Ulm University, Albert-Einstein-Allee 11, 89081 Ulm, Germany; 2 Institute of Immunology, Ulm University Medical Center, Albert-Einstein-Allee 11, 89081 Ulm, Germany; 3 Institute of Molecular Medicine, Ulm University, Albert-Einstein-Allee 11, 89081 Ulm, Germany; 4 Institute of Pathology, Ulm University Medical Center, Albert-Einstein-Allee 11, 89081 Ulm, Germany; 5 Center of Molecular and Cellular Oncology, Yale School of Medicine, 300 George Street, 06520 New Haven, CT USA

**Keywords:** Leukaemia, Cell division

## Abstract

The D-type cyclins (CCND1, CCND2, and CCND3) in association with CDK4/6 are known drivers of cell cycle progression. We reported previously that inactivation of FOXO1 confers growth arrest and apoptosis in B-ALL, partially mediated by subsequent depletion of CCND3. Given that previously the canonical MYC target CCND2 has been considered to play the major role in B-ALL proliferation, further investigation of the role of FOXO1 in CCND3 transcription and the role of CCND3 in B-ALL is warranted. In this study, we demonstrated that CCND3 is essential for the proliferation and survival of B-ALL, independent of the mutational background. Respectively, its expression at mRNA level exceeds that of CCND1 and CCND2. Furthermore, we identified FOXO1 as a CCND3-activating transcription factor in B-ALL. By comparing the effects of CCND3 depletion and CDK4/6 inhibition by palbociclib on B-ALL cells harboring different driver mutations, we found that the anti-apoptotic effect of CCND3 is independent of the kinase activity of the CCND3-CDK4/6 complex. Moreover, we found that CCND3 contributes to CDK8 transcription, which in part might explain the anti-apoptotic effect of CCND3. Finally, we found that increased CCND3 expression is associated with the development of resistance to palbociclib. We conclude that CCND3 plays an essential role in the maintenance of B-ALL, regardless of the underlying driver mutation. Moreover, downregulation of CCND3 expression might be superior to inhibition of CDK4/6 kinase activity in terms of B-ALL treatment.

## Introduction

B-cell lymphoblastic leukemia (B-ALL) is the most common pediatric neoplasia [1]. Pediatric B-ALL is associated with long-term survival of over 90%, whereas adult B-ALL shows dismal outcomes with cure rates below 40% [2]. Therapy in both children and adults typically consists of combinatory administration of cytostatic agents [3], which in many cases induce life treating toxicity and complications. Therefore, the search for novel, less toxic targeted therapies is warranted [4].

We reported previously that the tightly regulated expression of the transcription factor forkhead box protein O1 (FOXO1) is essential for the maintenance of B-ALL [5]. Given that FOXO1 had been identified as a canonical tumor suppressor, our finding was paradoxical [6]. However, our data were later corroborated in a BCR-ABL1-transformed B-­ALL mouse model with inducible *Foxo1* deletion [7]. Most importantly, we demonstrated that genetic and pharmacological inhibition of FOXO1 downregulates Cyclin D3 (CCND3) expression and the cytotoxic effects of FOXO1 depletion could be ameliorated by CCND3 overexpression [5].

The three D-cyclins CCND1, CCND2, and CCND3 work in a holoenzyme complex with the cyclin-dependent kinases CDK4 and CDK6. Upon mitogenic stimulation, the complex phosphorylates retinoblastoma protein 1 (RB1), which represses the transcriptional activity of E2Fs [8]. Following phosphorylation, RB1 is released, thereby permitting the E2F mediated initiation of the G1-S transcriptional program.

Throughout B-cell development, expression levels of CCND2 and CCND3 vary, while expression of CCND1 is stopped after differentiation from hematopoietic stem cells [9]. CCND3 plays a key role during the development of B-cell precursor cells and cannot be substituted by another D-type cyclin [9]. Additionally, at later stages of B-cell development, CCND3 is essential for the expansion of germinal center B-cells [10].

The role of CCND3 was extensively investigated in T-cell acute lymphoblastic leukemia (T-ALL). It was shown that *Ccnd3*^−/−^ mice are less susceptible to the development of T-ALL [11], and the ablation of *Ccnd3* in *Notch1*-driven T-ALL induces apoptosis [12]. In B-ALL however, the role of CCND3 has been neglected since in BCR-ABL1^+^ B-ALL it has been demonstrated that the proto-oncogene MYC canonically induces transcription of CCND2 [13], suggesting its role as the supreme D-cyclin in B-ALL [14].

Since our previous work on FOXO1-depletion indicated a major role of CCND3 in B-­ALL, we investigated the transcriptional regulation of CCND3 by FOXO1 and the effects of acute loss of CCND3 in B-ALL cells of different genetic backgrounds. In addition, we addressed the CDK4/6-independent role of CCND3.

## Results

### CCND3 is the highest expressed D-Cyclin in all subtypes of B-ALL

**Fig. 1:**
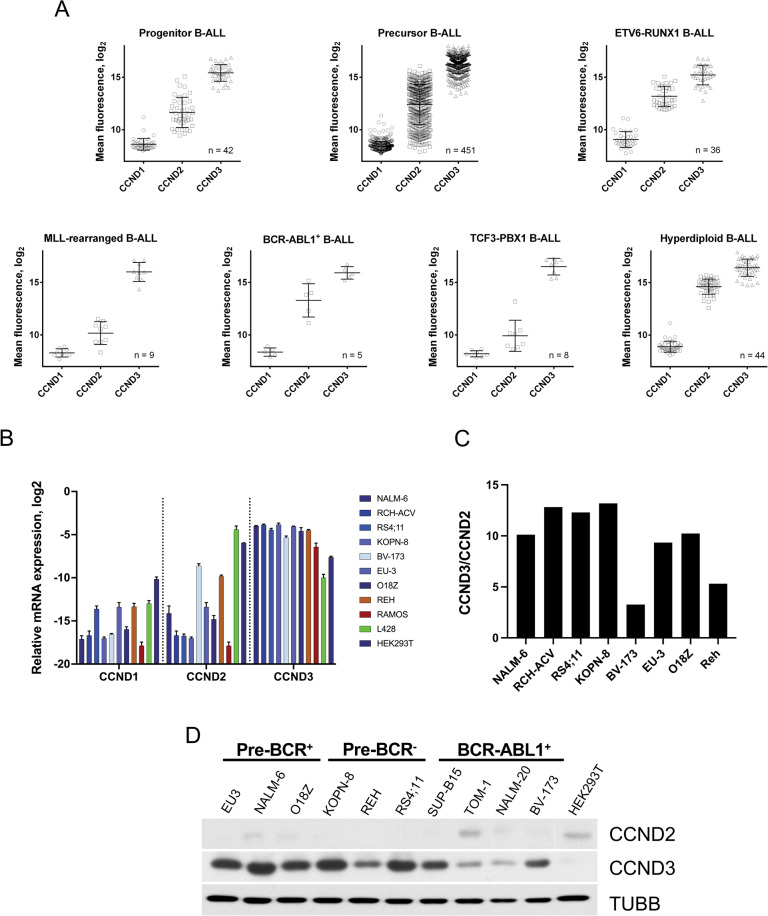
CCND3 exceeds expression of CCND1 and CCND2 at mRNA and protein levels in B-ALL. **A** The mRNA expression data of *CCND1*, *CCND2*, and *CCND3* in different B-ALL subtypes were mined from publicly available databases using the GENEVESTIGATOR software (http://www.genevestigator.com/). The experiment IDs are listed in Supplementary Materials and Methods. Data shown as mean ± SD. **B** The mRNA expression levels of D-type cyclins in B–ALL (blue) and control cell lines was measured by qRT-PCR. The control cell lines included Burkitt lymphoma (Ramos), cHL (L428), and HEK293T which express high levels of *CCND3*, *CCND2*, and *CCND1*, respectively. Data shown as mean ± SD, *n* = 3. **C** Ratio of mRNA levels *CCND3/CCND2* measured by qRT-PCR in B-ALL cell lines. **D** Expression of CCND2 and CCND3 proteins in B-ALL cell lines was measured by immunoblot. TUBB is used as loading control. Image representative of *n* = 3.

To clarify the role of D-type cyclins in B-ALL, we compared their expression at mRNA level in the most common B-ALL subtypes (Fig. 1A). Compared to all other indexed types of cancer, *CCND3* showed the highest expression levels in B-ALL (Supplementary Fig. 1). Furthermore, *CCND3* was expressed at much higher levels than *CCND1* and *CCND2* in all B-ALL subtypes. *CCND1* appeared to be the least expressed D-cyclin in all genetic groups of B-ALL. To clarify whether the observed differences in the expression levels of D-type cyclins indicate the exceptional functional significance of CCND3, we used publicly available data of genome-wide CRISPR/Cas9 loss-of-function viability screenings (https://depmap.org/portal/). For all indexed B-ALL cell lines (SEM, SEMK2, 697, RCH-ACV, REH, JM-1, NALM-6, HB1119, and P300HK), estimated dependency on CCND3 was much higher than for CCND1 and CCND2 (Supplementary Fig. 2A, B).

Next, we asked whether the high CCND3 expression is maintained in human B-ALL cell lines. To this end, we measured mRNA and protein expression levels of the D-cyclins in B-ALL cell lines harboring different mutations (Fig. 1B,D). The ratio of *CCND3*/*CCND2* mRNA expression in all cell lines was similar to the primary B-ALL samples (Fig. 1C). *CCND2* expression varies throughout genotypes with higher expression in BCR-ABL1^+^ cell lines. CCND1 was not detectable on protein level in any B-ALL cell line (Supplementary Fig. 3).

This data indicates that CCND3 is by far the highest expressed D-type cyclin in B-ALL patient samples and cell lines, independent of the underlying driver mutation.

### CCND3 transcription is directly regulated by FOXO1

**Fig. 2:**
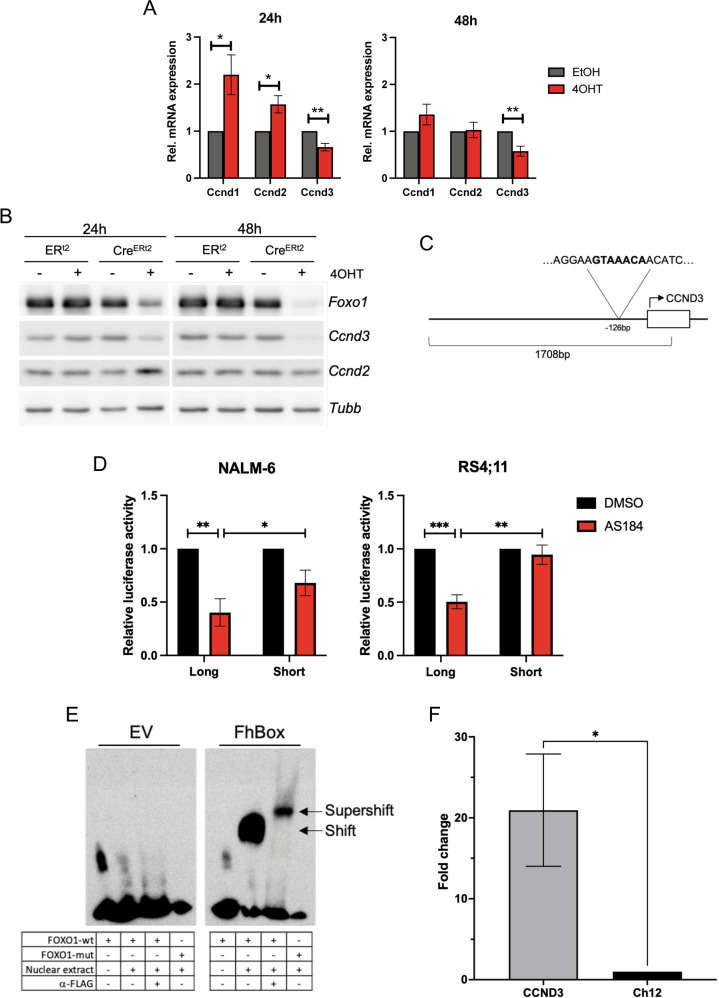
FOXO1 regulates CCND3 transcription by DNA binding. **A** Deletion of *Foxo1* in BCR-ABL1^+^ murine B-ALL cells decreases mRNA and **B** protein expression of *Ccnd3* but increases *Ccnd1* and *Ccnd2* mRNA expression. *Ccnd1* was not detectable on protein level. Data shown as mean ± SD, *n* = 3. Statistical analysis was performed with students t-test. *=*p* < 0.05 **=*p* < 0.01. **C** *CCND3* promoter region −1389 to +259 bp from the *CCND3* TSS (NC_000006.12, https://www.ncbi.nlm.nih.gov, 22 May 2020) used for luciferase assay containing the FOXO binding motif GTAAACA −126 bp from the transcriptional start site. **D** NALM-6 and RS4;11 cells were co-transfected with either pGL4.22-*CCND3*-promoter (long) or a truncated version containing the core promoter without the FOXO binding motif (short), together with Ubi-*Renilla* luciferase-expressing vectors and treated for 24 h with the FOXO1 inhibitor AS1842856 at a concentration of 80 nM. The luminescence intensities of the experimental vectors were normalized to Ubi-*Renilla*. Data shown as mean ± SD, *n* = 3. Statistical analysis was performed with students t-test. *=*p* < 0.05 **=*p* < 0.01. ***=*p* < 0.005 **E** Binding of FOXO1 to the *CCND3* promoter (NC_000006.12, https://www.ncbi.nlm.nih.gov, 22 May 2020) shown by EMSA. Nuclear extracts from HEK293T cells transfected with either pFLAG-CMV2-Empty Vector (EV) or pFLAG-CMV2-FhBox (FhBox) were co-incubated with DNA probes containing FOXO binding motif −126 to −101 bp from the *CCND3* TSS (FOXO1-wt) or scrambled FOXO binding motif (FOXO1-mut). Image representative of *n* = 2. **F** Chromatin immunoprecipitation. NALM-6 cells transfected with humanized biotin ligase BirA and either a constitutively active variant of FOXO1 with N-terminal biotinylation signal or empty control vector. Pulldown was performed with magnetic streptavidin particles. Enrichment of the DNA fragment containing the FOXO binding motif (CCND3) was analyzed by qRT-PCR with two primers flanking the binding motif. Ct values were first normalized to the respective input and then calculated relative to the signal obtained by amplification with primers targeting the Ch12 gene desert (Ch12). Data are shown as mean ± SD, *n* = 3. Statistical analysis was performed with students t-test. *=*p* < 0.05.

FOXO1 was shown to repress the transcription of *CCND1* and *CCND2* [15]. At the same time, FOXO1 activates *Ccnd3* transcription in murine pancreatic cells [16]. Moreover, we have shown that FOXO1 inactivation decreases CCND3 expression in human B-ALL cell lines [5]. To further investigate the dependency of *CCND3* transcription on FOXO1, we induced depletion of *Foxo1* in a BCR-ABL1^+^ *Foxo1*^fl/fl^ murine B-ALL model (Fig. 2A). This exclusively decreased *Ccnd3* but not *Ccnd1* or *Ccnd2* at mRNA and protein levels (Fig. 2B). In line with the known role of FOXO1 as a negative regulator of *Ccnd1* and *Ccnd2* transcription, both Cyclins were significantly upregulated on mRNA level after 24 h. Furthermore, 24 h after *Foxo1* deletion, CCND2 was upregulated on protein level. However, this upregulation of CCND2 could not substitute CCND3 in the maintenance of cell cycle progression (Supplementary Fig. 4).

Next, we addressed the mechanism underlying the decrease of *Ccnd3* transcription induced by *Foxo1* depletion. Previously we reported on the decrease of MYC transcription and RB1 phosphorylation by FOXO1 depletion in human B-ALL cell lines [5]. Given that both MYC as well as the RB1 repression target E2F1 might activate *CCND3* transcription [8, 17, 18], we investigated their role in the observed *Ccnd3* downregulation.

We found that *Foxo1* deletion decreased *Myc* transcription in the BCR-ABL1-transformed murine pre-B-cells (Supplementary Fig. 5A). Given that BCR-ABL1 activates *Myc* transcription and increases the inactivating phosphorylation of FOXO1 in this B-ALL model [7], we treated the cells with 1 µM of the BCR-ABL1 inhibitor imatinib to abolish *Myc* expression. Imatinib strongly decreased MYC expression at mRNA and protein levels (Supplementary Fig. 5B,C), but simultaneously increased FOXO1 protein level and reduced the FOXO1-inactivating S256 phosphorylation. *Ccnd3* mRNA expression remained stable. These data indicated that *Ccnd3* transcription does not depend on *Myc*. To address the role of E2Fs in *Ccnd3* transcription [18], we treated the cells with the CDK4/6 inhibitor palbociclib at an effective concentration of 1 µM, which completely arrested cell cycle progression in human B-ALL cells. Palbociclib increased FOXO1 protein expression (Supplementary Fig. 6A) and concomitantly increased *Ccnd3* expression at mRNA (Supplementary Fig. 6B) and protein levels (Supplementary Fig. 6C) suggesting that also E2Fs are not essential for *Ccnd3* transcription in B-ALL.

We further investigated the effect of FOXO1 on the activity of the *CCND3* promoter using a luciferase-reporter assay. To this end we transfected NALM-6 and RS4;11 B-ALL cells with a reporter construct driven by the *CCND3* promoter spanning from −1389 to +259 bp from the transcriptional start site (TSS) and harboring the FOXO binding motif GTAAACA −126 bp from the TSS (“long”) [19] (Fig. 2C). As a control, we used the core promoter region of *CCND3*, which lacks the FOXO binding motif (“short”). Treatment with the FOXO1 inhibitor AS1842856 at a concentration of 80 nM significantly decreased the activity of the “long” but not the “short” reporter construct (Fig. 2D).

To further elaborate on the transcriptional regulation of *CCND3* by FOXO1, we analyzed the binding of the forkhead DNA-binding domain (FhBox) to the FOXO-binding site located −126 bp from the *CCND3* TSS. To this end, we performed electrophoretic mobility shift assay (EMSA). Nuclear extracts of HEK293T cells transfected with either FhBox-FLAG or empty vector (EV) were co-incubated with probes corresponding to −142 to −117 bp from the TSS and containing the FOXO binding motif. The construct containing the FOXO-binding motif was only shifted by the nuclear extracts expressing FhBox, but not by the control extracts (Fig. 2E and Supplementary Fig. 7A). Mutation of the FOXO binding motif completely abolished the FhBox binding. The specificity of the binding of FhBox to the FOXO binding site was confirmed by super-shift assay.

In order to confirm the direct binding of FOXO1 at the *CCND3* promoter, we first mined available ChIP-seq data on binding of FOXO1 to transcription regulatory elements in pre-leukemic stem cells and mature B-lymphocytes (Supplementary Fig. 8). Both sample tracks showed significant binding events at the *CCND3* promoter in the proximity of the FOXO binding site which we identified by EMSA. Next, we experimentally corroborated FOXO1 binding to the *CCND3* promoter with help of ChIP-qRT-PCR. To this end we used a constitutively active variant of FOXO1, containing an N-terminal biotinylation signal, as we described previously (Supplementary Fig. 7B) [20].

Using this approach, we demonstrated direct binding of FOXO1 at the *CCND3* promoter (Fig. 2F).

### CCND3 is essential for the growth and survival of B-ALL cells

**Fig. 3:**
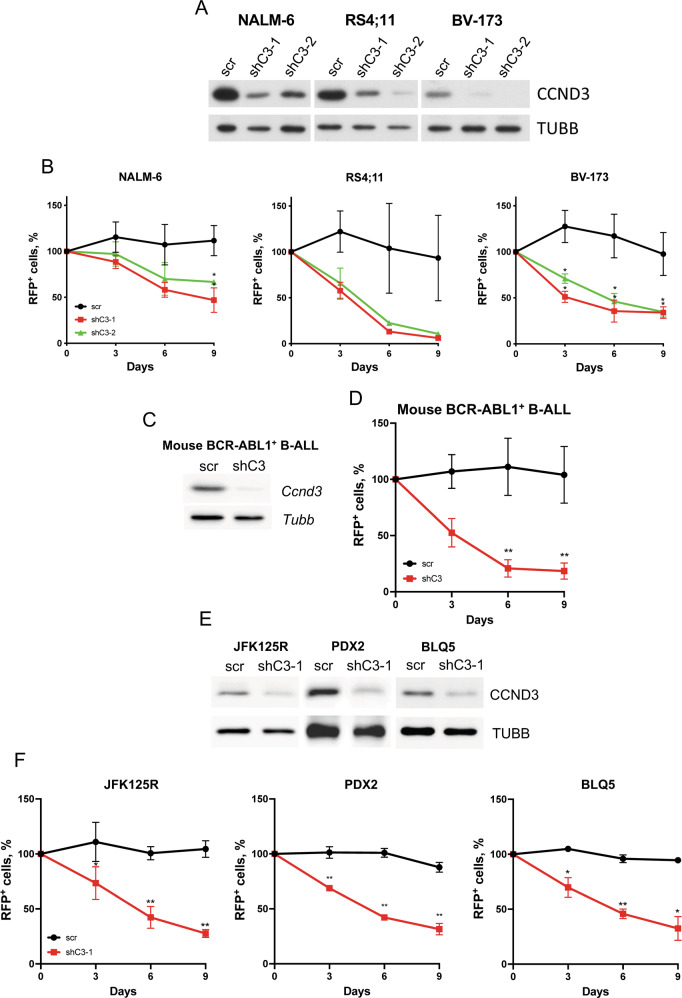
Downregulation of CCND3 is toxic for B-ALL cells. **A** Efficiency of the shRNA-dependent *CCND3* knockdown was assessed by immunoblot. Image is representative of *n* = 3. **B** B-ALL cell lines were transduced with plasmids expressing RFP and either a non-targeting control, or *CCND3*-targeting shRNAs. Data shown as mean ± SD, *n* = 3. Statistical analysis was performed with student’s t-test. *=*p* < 0.05. **C** Immunoblot shows downregulation of CCND3 expression by specific shRNA in BCR-ABL1 transformed mouse pre-B-cells. Image representative of *n* = 3. **D** shRNA-dependent knockdown of *Ccnd3* in BCR-ABL1 transformed murine pre-B-cells significantly inhibits their fitness in a competitive growth assay. Data shown as mean ± SD, *n* = 3. Statistical analysis was performed by student’s t-test. **=*p* < 0.01. **E** B-ALL PDXs JFK125R, PDX2, and BLQ5 were transduced with vectors expressing a shRNA targeting *CCND3*. CCND3 downregulation was analyzed by immunoblot. Image representative of *n* = 3. **F** The cytotoxic effect of CCND3 depletion was analyzed by competitive growth test as described in legend to Fig. 3B. Data shown as mean ± SD, *n* = 3. Statistical analysis was performed by student’s t-test. *=*p* < 0.05 **=*p* < 0.01.

Previously we have shown that CCND3 knockdown induces growth arrest and apoptosis in the B-ALL cell lines NALM-6 and RS4;11, which harbor ETV6-PDGFR [21] and MLL-AF4 [22] translocations, respectively. Here we set to investigate the role of CCND3 in the oncogenic program of other B-ALL subtypes, including BCR-ABL1^+^ B-ALL cell lines, which are distinguished by the highest CCND2 expression compared to other genotypes. To deplete CCND3, we used two shRNAs. Both shRNAs efficiently downregulated CCND3 (Fig. 3A) and conferred a significant growth disadvantage compared to control cells transduced with a non-targeting construct in a competitive growth assay in all cell lines (Fig. 3B and Supplementary Fig. 9A). This effect of shRNA-dependent knockdown was corroborated by CRISPR/Cas9-editing (Supplementary Fig. 9B, C).

The pro-apoptotic and growth-inhibitory effect of CCND3 knockdown was also observed in an ex vivo BCR-ABL1^+^ murine B-ALL model with a shRNA construct targeting *Ccnd3* (Fig. 3C, D). Additionally, we corroborated our data on the essential role of CCND3 by including three B-ALL patient-derived xenografts (PDX). JFK125R, PDX2, and BLQ5. JFK125R represents refractory, *KRAS*^G12D^-driven B-ALL, PDX2 harbors the BCR-ABL1 translocation and *IKZF1*-deletion, and BLQ5 was derived from a relapsed BCR-ABL1^+^ B-ALL patient after treatment with imatinib [23]. In all three PDXs, *CCND3* knockdown was detrimental to survival.

We conclude that CCND3 is essential for the survival of B-ALL independently of the type of driver mutation.

### The pro-apoptotic effect of CCND3 downregulation is independent of CDK4/6 kinase activity

**Fig. 4:**
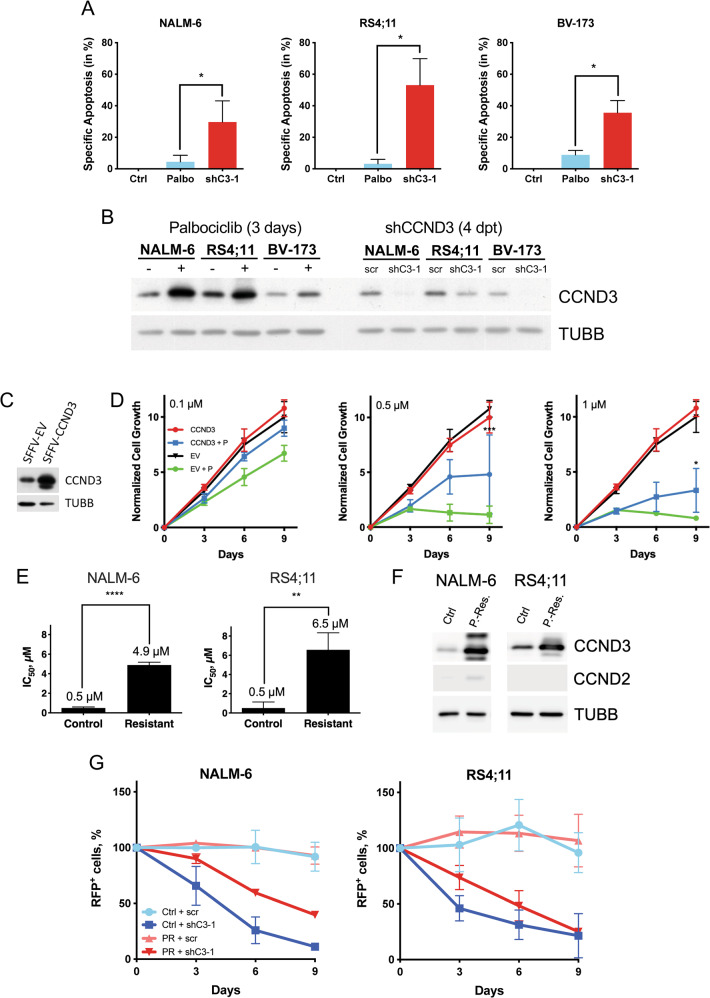
CCND3 protects B-ALL from apoptosis in a kinase-independent manner and confers resistance to palbociclib. **A** B-ALL cell lines were treated with 1 µM palbociclib for 3 days or transduced with either a scrambled (control) or a CCND3-shRNA containing vector, harboring a fluorescent marker (RFP). The RFP^+^ cells were sorted by FACS 4 days post transduction and cultured for 3 more days. Apoptosis was analyzed by PI/annexin V staining, with help of flow cytometry. Data shown as mean ± SD, *n* = 3. Statistical analysis was performed by student’s t-test. *=*p* < 0.05 **B** CCND3 protein expression level was analyzed by immunoblot 3 days after treatment with 1 µM palbociclib and 4 days after transduction with either scrambled control or CCND3 targeting shRNA. Image is representative of *n* = 3. **C** NALM-6 cells were transduced with SFFV-CCND3 vector expressing wtCCND3 and a fluorescent marker (EGFP) or by control vector (EV). CCND3 overexpression was controlled by immunoblot. **D** Cell growth of CCND3 overexpressing NALM-6 cells and EV control expressing NALM-6 cells was determined by calculating GFP^+^ cells relative to the absolute cell number of living cells. Data shown as mean ± SD, *n* = 3. Statistical analysis was performed by Student’s t-test. *=*p* < 0.05, ***=*p* < 0.005. **E**, **F** Cells were cultured in the presence of increasing (from 0.01 to 1.7 µM) concentrations of palbociclib for 3 months. After reaching 1.7 µM concentration with cells growing at a speed comparable to the vehicle-treated control cells, their sensitivity to palbociclib was measured using MTT Assay (**E**) and CCND3 and CCND2 expression at the same time point was measured by immunoblot (**F**). Image is representative for *n* = 3. Statistical analysis was performed by student’s t-test. **=*p* < 0.01, ****=*p* < 0.001. **G** Control (Ctrl) and palbociclib-resistant (PR) NALM-6 and RS4;11 cells were transduced with either a scrambled shRNA (scr) or shRNA against *CCND3* (shC3), co-expressing RFP. The proportion of RFP^+^ cells was monitored throughout 9 days, starting 4 days post transduction (Day 0). Data shown as mean ± SD, *n* = 3.

In T-ALL cell lines, the anti-apoptotic effect of CCND3 depends on the kinase activity of the CCND3-CDK6 complex [24]. To clarify if the same holds true in B-­ALL, we treated the B-ALL cell lines NALM-6, RS4;11 and BV-173 with the highly specific CDK4/6 inhibitor palbociclib at a concentration of 1 µM, which has been shown to induce apoptosis in other B-and T-cell malignancies [25], and was approximately two-fold higher than the maximally achievable plasma concentration in patients [26]. Both palbociclib and *CCND3* knockdown significantly decreased the number of cells in S-phase in NALM-6, RS4;11, and BV-173 cell lines (Supplementary Fig. 10). However, only *CCND3* knockdown but not treatment with palbociclib induced strong apoptosis (Fig. 4A). Induction of apoptosis by *Ccnd3* knockdown was also confirmed in an ex vivo BCR­ABL1^+^ mouse B-ALL model (Supplementary Fig. 11).

We concluded that other mechanisms than inhibition of CDK4/6 kinase activity are responsible for the pro-apoptotic effect of CCND3 depletion in B-ALL.

### Increase of CCND3 expression contributes to the resistance to palbociclib

We noticed that short treatment with palbociclib strongly increased the expression of CCND3 protein in all cell lines (Fig. 4B). CCND1 protein remained undetectable in all cell lines, whereas CCND2 was slightly upregulated in NALM-6, not detectable in RS4;11 and increased in BV-173 (Supplementary Fig. 12A). In order to assess if the upregulation of CCND3 represents an adaptation mechanism, we overexpressed CCND3 in NALM-6 cells (Fig. 4C) and incubated them in presence of increasing concentrations of palbociclib (Fig. 4D). Ectopic CCND3 expression did not affect the proliferation parameters of control cells, but significantly decreased sensitivity of NALM-6 to the growth-inhibitory effects of palbociclib.

In order to interrogate whether CCND3 upregulation poses a mechanism for acquired resistance to palbociclib, NALM-6 and RS4;11 were cultured in the presence of gradually increased concentrations of palbociclib. In due course of the selection, the IC_50_ of palbociclib increased from 0.5 to 4.9 µM in NALM-6 and from 0.5 to 6.9 µM in RS4;11, respectively (Fig. 4E). The palbociclib-resistant NALM-6 and RS4;11 cells showed highly elevated expression levels of CCND3 and FOXO1, much higher than after short-term treatment (Fig. 4F and Supplementary Fig. 12B). CCND2 protein was slightly increased in resistant NALM-6 but not detectable in RS4;11 (Fig. 4F). Importantly, B-ALL cells resistant to palbociclib were still sensitive to the growth-inhibitory effect of CCND3 knockdown (Fig. 4G).

Thus, upregulation of CCND3 contributes to the acquisition of resistance to palbociclib in B-ALL.

### CDK8 repression contributes to the cytotoxic effects of *CCND3* knockdown

**Fig. 5:**
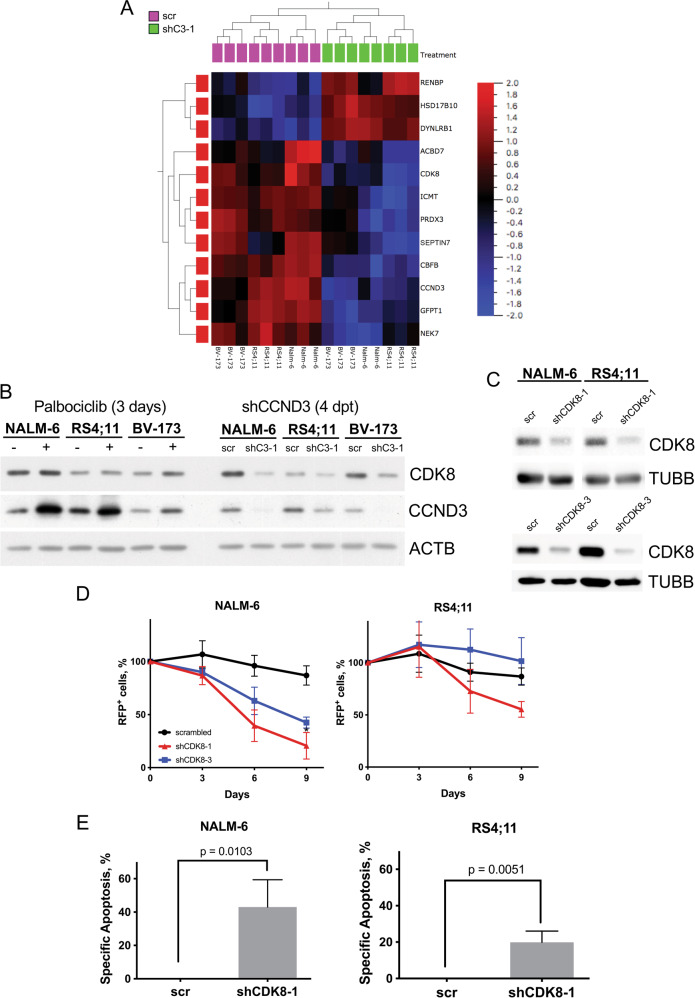
CDK8 downregulation contributes to the cytotoxicity induced by CCND3-knockdown. **A** Heatmap generated from RNA-Seq data performed for the three B-ALL cell lines NALM-6, RS4;11 & BV-173, which were either treated with 1 µM palbociclib for 3 days or sorted after 4 days post lentiviral transduction with either a shRNA targeting *CCND3* (shC3-1) or control vector (scr). The heatmap represents the result of unsupervised hierarchical clustering of the 12 genes that were exclusively regulated only by *CCND3*-knockdown in all cell lines. (Supplementary Fig. 13). *q* = 0.1, *p* < 0.05. **B** CDK8 protein levels are downregulated by *CCND3*-knockdown, not by palbociclib treatment. CDK8 levels were analyzed by immunoblot either after three days of 1 µM palbociclib treatment or four days post transduction with a *CCND3*-targeting shRNA. *n* = 3, image representative. **C** ShRNA-dependent *CDK8* knockdown was controlled by immunoblot, image representative of *n* = 3. **D** Both *CDK8*-targeting shRNAs reduce cell fitness in a competitive growth assay (see legend to Fig. 3B). Data shown as mean ± SD, *n* = 3. **E** CDK8-knockdown by shRNA induces significant apoptosis in B-ALL cell lines. Cells were transduced as described in the legend of Fig. 5D and sorted four days after transduction for 100% RFP^+^ cells, then cultured for two more days. Specific apoptosis was determined by staining with PI and for Annexin-V, measured via flow cytometry. Data shown as mean ± SD, *n* = 3 Statistical analysis was performed by Student’s t-test.

To identify mechanisms responsible for the CDK4/6 kinase-independent anti-apoptotic function of CCND3, we compared the effects of palbociclib and *CCND3* knockdown at transcriptome level in three B-ALL cell lines of different subtypes using RNA-sequencing (Supplementary Table S1). Using hierarchical clustering and Venn analysis, we identified genes differentially regulated by *CCND3* knockdown and palbociclib treatment throughout all three cell lines (Fig. 5A and Supplementary Fig.13). Among the genes repressed only by *CCND3* knockdown were genes regulating metabolism (*ABCD7* [27], *RENBP* [28], *SEPTIN7* [29]), oxidative stress (*PRDX3* [30], *HSD17B10* [31]) and genes involved in oncogenic processes *(NEK7* [32], *DYNLRB1* [33], *ICMT* [34], *CBFB* [35], *GFPT1* [36], *CDK8* [37]). Given that the non-canonical kinase CDK8 was recently identified as an essential transcriptional regulator in BCR-ABL1^+^ B-ALL [37], we focused on this gene. Since the role of CDK8 was investigated in detail only in the oncogenic program of BCR-ABL1^+^ B-ALL, we addressed the function of CDK8 in other B-­ALL subtypes. Using immunoblot, we corroborated CDK8 protein downregulation by CCND3 knockdown but not by treatment with palbociclib in all three cell lines (Fig. 5B).

Since it has been shown that loss of CDK8 protein but not inhibition of its enzymatic activity is cytotoxic for BCR­ABL1^+^ B-ALL [37], we assessed the sensitivity of NALM-­6 and RS4;11 cells to the CDK8 inhibitor SEL120. Both cell lines did not differ in sensitivity to SEL120 compared to the non-sensitive BCR-ABL1^+^ cell line BV­-173 (Supplementary Fig. 14) [37].

To investigate whether loss of CDK8 protein is detrimental to B-ALL cells regardless of the underlying genotype, we repressed *CDK8* in NALM-6 and RS4;11 by using two shRNAs (Fig. 5C). Depletion of *CDK8* significantly decreased the fitness of cells in a competitive growth assay (Fig. 5D). Importantly, *CDK8* knockdown induced apoptosis in both cell lines (Fig. 5E).

We concluded that CCND3 regulates *CDK8* transcription and that downregulation of CDK8 might contribute to the anti-apoptotic effect of CCND3 in B-ALL.

## Discussion

We demonstrated that CCND3 is indispensable for the maintenance of B-ALL, regardless of the underlying oncogenic genotype. The anti-apoptotic effect of CCND3 did not depend on the catalytic activity of the CCND3-CDK4/6 complex. Furthermore, we identified a role of CCND3 in the transcription of the anti-apoptotic kinase CDK8 and revealed the role of FOXO1 in the regulation of *CCND3* transcription.

We found that CCND3 is the highest expressed D-cyclin throughout all major subtypes of B-ALL. Moreover, the data of genome wide loss-of-function screening indicate the superior role of CCND3 specifically in oncogenic programs of B-ALL subtypes. This is surprising, since so far, CCND2 has widely been considered to be the most important D-cyclin during B-cell development and in B-ALL. CCND2 is a direct transcriptional target of the proto-oncogene MYC, a key driver of B-lymphoblast expansion and oncogenic signaling in B-ALL [38]. CCND2 is abundant throughout all stages of early B-cell development [9]. In non-neoplastic pro- and pre-B-cells, survival signals converge in the activation of MYC transcription and its stabilization.

In B-ALL, the essential role of MYC was especially well studied in BCR-ABL1^+^ cases [14]. The BCR-ABL1 oncogene activates transcription only of *Ccnd2* via MYC, but not *Ccnd3* as it has been shown in murine pre-B-cells [7]. Indeed, our analysis has demonstrated that BCR-ABL1^+^ B-ALL expressed higher levels of CCND2 than other subtypes.

CCND1 has been shown to be expressed in very low levels in pro- and pre-B-cells [9]. Indeed, our analysis demonstrated that this carries over to their malignant counterparts, since no B-ALL cell line showed detectable CCND1 protein expression. Overall, even in BCR-ABL1^+^ cases, CCND3 was always the highest expressed D-cyclin.

Importantly, by using loss-of-function experiments we demonstrated an essential role of CCND3 in cell lines and PDXs representing different subtypes of B-ALL including BCR-ABL1^+^ ones. The observed cytotoxic effect of CCND3 depletion indicates a unique role of CCND3, which depletion cannot be compensated by the relatively high CCND2 levels of BCR-ABL1^+^ B-ALL.

We demonstrated that apoptosis in B-ALL cell lines can only be efficiently induced by genetic depletion of *CCND3* but not by inhibition of the CCND3-associated kinases CDK4/6 by palbociclib. This finding indicates principal differences of B-ALL to T-ALL, where palbociclib induces apoptosis by repressing the pentose phosphate pathway, thereby inducing oxidative stress [24]. Our transcriptome analysis did not reveal enrichment of the oxidative stress signature neither after treatment with palbociclib nor after CCND3 knockdown. Interestingly, although it was documented that palbociclib is not able to induce apoptosis in B-ALL, genetic depletion, or a PROTAC-dependent degradation of CDK6, the predominantly expressed CDK that forms a holoenzyme with CCND3, induces cell death in BCR­ABL1^+^ B-ALL. This, together with our findings, indicates kinase-independent effects of the components of the CCND-CDK holoenzyme [39].

We conclude that CCND3 indeed possesses unique, non-canonical, essential functions in B-ALL that cannot be substituted by another D-type cyclin, as previously suggested by others [40, 41].

Comparing the transcriptome changes induced by *CCND3* knockdown versus palbociclib, we identified *CDK8* among the genes exclusively repressed by *CCND3* knockdown and demonstrated that knockdown of *CDK8* induces apoptosis in B-ALL cell lines harboring ETV6-PDGFRB and MLL-AF4 translocations. It has been demonstrated that the pro-survival effects of CDK8 include facilitation of glycolysis, activation of STAT1 and STAT5 oncogenic signaling, and activation of mTORC1 [37]. The sensitivity of BCR-ABL1^+^ B-ALL to genetic and PROTAC-mediated CDK8 depletion has been already shown to particularly depend on subsequent inactivation of mTORC1 activity [37]. Our data indicate a role of CCND3 in the regulation of *CDK8* transcription and show that the anti-apoptotic effect of CDK8 is not limited to the BCR-ABL1^+^ subtype.

Although CCND3 is mostly known as a canonical component of the CCND-CDK4/6 holoenzyme complex, which inactivates RB1 and thereby activates E2F-dependent transcription, this mechanism most possibly is not involved in the regulation of *CDK8* transcription. Even relatively high concentrations of palbociclib which we used did not influence *CDK8* expression. At the same time, there is much evidence on the involvement of CCND3 in the regulation of transcriptional activity independently of E2Fs. In particular, CCND3 binds to the nuclear matrix, restricting access to multiple genes including the V*k* locus [41, 42]. CCND3 can also regulate the activity of transcription factors such as Androgen Receptor (AR) or RUNX1 by direct binding to them, thereby modulating their activity [43, 44].

Thus, direct transcriptional regulation might be instrumental in the anti-apoptotic effect of CCND3 in B-ALL.

We identified increased *CCND3* expression as an adaptation mechanism facilitating the development of resistance to palbociclib treatment. The increase of CCND3 protein expression after treatment with palbociclib might in part be explained by a paradoxical stabilization of CCND3-CDK4/6 complexes [45]. However, we observed an increase of CCND3 expression not only at protein, but also at mRNA levels, indicating involvement of transcriptional mechanisms.

Interestingly, in a *KRAS*-driven non-small cell lung cancer mouse model, ERK-induced concomitant upregulation of *Ccnd1*, as well as *Ccnd3*, was observed, rendering the tumor resistant to palbociclib [46]. Taking this into account, we cannot exclude that the observed increase of CCND2 protein in BV-173 after treatment with palbociclib represents an additional adaptation mechanism. In T-ALL, upregulation of CCND3 after treatment with palbociclib was also observed, albeit not further explained [24].

We identified FOXO1 as a direct transcriptional activator of *CCND3*. This finding is not absolutely unexpected, since FOXO1 has been shown to directly bind to the *Ccnd3* promoter and activate its transcription in murine pancreatic cells [16]. In early B-cells, PAX5 has been considered as a main activator of *CCND3* transcription [47]. Interestingly, FOXO1 is a negative regulator of PAX5 in healthy B-cell precursors, inhibiting proliferation and inducing differentiation [48].

On the contrary, the direct FOXO1 transcription target *EBF1*, which, together with FOXO1 comprises a positive feedback loop, also activates *CCND3* transcription [49]. This is in line with ours and others data, showing the essential role of FOXO1 for the maintenance and proliferation of B-ALL [5, 7], and in pro-B-cells where FOXO1 knockdown induces growth arrest and apoptosis [50].

Apart from PAX5 and EBF1, MYC and E2F might also activate *CCND3* transcription [18, 51]. However, our functional analysis showed that inhibition of neither MYC with imatinib nor E2F with palbociclib negatively impacted *CCND3* transcription, as long as FOXO1 is present, which was upregulated by both treatments.

Of note, unstimulated germinal center B-cells express high levels of FOXO1 as well as CCND3, but both are downregulated upon stimulation of the BCR, simultaneously with MYC upregulation. Moreover, forced activation of MYC and thereby activation of *CCND2* transcription was not able to overcome the growth arrest after *CCND3* deletion in germinal center B-cells [52].

Taken together, we conclude that although transcriptional regulation of *CCND3* expression is complex and requires cooperative interaction of multiple factors, involvement of FOXO1 is essential for complete *CCND3* transcription, particularly in B-ALL.

Conclusively, we demonstrated an essential role of CCND3 in the maintenance of B-ALL of major genetic subtypes using different in vitro and ex vivo models. Moreover, we revealed the mechanistic basis of CCND3 downregulation after FOXO1 depletion by showing direct binding of FOXO1 to the *CCND3* promoter, activating its transcription. Furthermore, we revealed that the anti-apoptotic effect of CCND3 does not depend on kinase activity. Lastly, we identified CDK8 as an anti-apoptotic, transcriptionally regulated target of CCND3, providing further incentive for therapeutic intervention by CDK8 degradation or transcriptional inhibition.

## Materials and methods

Additional and detailed information on all experimental procedures and reagents is provided in Supplementary Materials and Methods.

### Data mining

Publicly available RNA-expression data were mined by using GENEVESTIGATOR software. Experiment IDs are listed in Supplementary Table 1.

### Cell culture

B-ALL cell lines NALM-6, RS4;11, BV-173, REH, TOM-1, NALM-20, SUP-B15, KOPN-8, and EU-3/697, the classical Hodgkin lymphoma (cHL) cell line L428 and Burkitt lymphoma cell line RAMOS, HEK293T, and LentiX cells were purchased from DSMZ (Braunschweig, Germany). The B-ALL cell line 018Z was provided by Meyer L-H., (Ulm University, Germany). Details for the human cell lines used are listed in Supplementary Table 2. Cre-ER^T2^ and ER^T2^ murine pre-B-cells homozygous for *loxP* flanked *Foxo1* (*Foxo1*^fl/fl^) transformed with BCR-ABL1 were a gift of Jumaa H. (Ulm University, Germany) [7]. The B-ALL patient-derived xenografts (PDX) JFK125R, PDX2, and BLQ5 were a kind gift of Müschen M. (Yale School of Medicine, USA).

### Immunoblot and qRT-PCR

Immunoblot and qRT-PCR were done as described previously [5]. Primers used for qRT-PCR are listed in Supplementary Table 3. Antibodies used for immunoblot are listed in Supplementary Table 4.

### Luciferase assay, EMSA, and ChIP

Primers used for cloning of the luciferase reporter constructs are listed in Supplementary Table 5. Oligos used for EMSA are listed in Supplementary Table 6. Primers for qRT-PCR analysis of ChIP are listed in Supplementary Table 7.

### Vectors and lentiviral transduction

Lentiviral transduction was done as described previously [5]. Sequences used for cloning of the shRNA constructs are listed in Supplementary Table 8. CRISPR/Cas9 gRNA sequences are listed in Supplementary Table 9.

### Flow cytometry, cell sorting, cell cycle, and apoptosis measurement

Growth dynamics, cell sorting, cell cycle analysis, and apoptosis measurement of lentivirally transduced and treated cells were performed as described previously [5].

### IC_50_ determination

The sensitivities of the cell lines to the CDK4/6 inhibitor palbociclib and the CDK8 inhibitor SEL120 were assessed by MTT assay as we described previously [5].

### Data analysis

Data were analyzed by two-tailed Student’s *t*-test analysis and by one-way ANOVA using GraphPad PRISM (GraphPad Software, San Diego, CA).

## Supplementary information

Supplementary Information

Supplementary Table S1

## Data Availability

The RNA-sequencing datasets generated and analyzed in this study are available at NCBI GEO database (https://www.ncbi.nlm.nih.gov/), accession number GSE178660.
