# Supplementary material for: CCND3 is indispensable for the maintenance of B-cell acute lymphoblastic leukemia
Source: Oncogenesis. 2022 Jan 10;11(1):1. doi: 10.1038/s41389-021-00377-0 (PMC8748974; doi:10.1038/s41389-021-00377-0)
Supplement: Supplementary file 1 — Supplementary Information [file 41389_2021_377_MOESM1_ESM.pdf]

Supplementary information related to:

CCND3 is indispensable for the maintenance of B-cell acute lymphoblastic leukemia

Authors:

Franz Ketzer, Hend Abdelrasoul, Mona Vogel, Ralf Marienfeld, Markus Müschen, Hassan Jumaa, Thomas Wirth*, Alexey Ushmorov*

Contents:

1. Supplementary Materials and Methods
2. Supplementary Figures

Supplementary Materials and Methods

Analysis of publicly available Gene expression profiling (GEP) data

GEP data were mined and analyzed using GENEVESTIGATOR on-line software (1) (RRID:SCR_002358) (Nebion AG, Zurich, Switzerland). The analyzed experiments are listed in Supplementary Table 1.

B-ALL subtype	Experiment ID
**Progenitor B-ALL**	HS-00636
**Precursor B-ALL**	HS-01387, HS-01385, HS-00730, HS-00719, HS-00636, HS-00635, HS-00430, HS-00240
**ETV6-RUNX1**	HS-01387, HS-01385
**MLL-rearranged**	HS-01385, HS-01387
**BCR-ABL1^{+}**	HS-01385, HS-01387
**TCF3-PBX1**	HS-01385, HS-01387
**Hyperdiploid**	HS-01385, HS-01387

Supplementary Table 1: Experiment IDs of analyzed GEP data sets

ChIP-seq data

Publicly available ChIP-sequencing data for FOXO1 in pre-leukemic stem cells (GEO accession: GSM2136846) and B-lymphocytes (GEO accession: GSM1668935) was mined from https://chip-atlas.org (accessed on August 27th, 2021). The analysis was performed using Integrated Genome Viewer (IGV, v. 2.10.3) and Genepalette (v.2.1.1).

B-ALL cell lines and PDXs

B-ALL cell lines (Supplementary Table 2) were cultured in RPMI1640 (Gibco Life Technologies, Carlsbad, California, USA) supplemented with 20 % fetal bovine serum (FBS), 2 mM L-glutamine, 100 U/mL penicillin and 100 µg/mL streptomycin at 37 °C and 5 % CO_2. Cell lines were authenticated by short tandem repeat (STR) DNA typing using GenomeLab™ GeXP Genetic Analysis System (Sciex, Darmstadt, Germany) and GenomeLabHuman STR primer set (Beckman Coulter, Brea, CA, USA) or experiments were performed right after receipt from DSMZ. STR profiles were

analyzed using the “Online STR Analysis” tool provided by DSMZ (comprehensive DSMZ database of STR cell line profiles, www.dsmz.de) and the ExPASy bioinformatics resource portal database “Cellosaurus” (https://web.expasy.org/cellosaurus/; RRID: SCR_013869). Cell lines were regularly tested for mycoplasma contamination using MycoAlert™ Mycoplasma Detection Kit (Lonza Group Ltd., Basel, Switzerland).

Cell line	Genetic Alteration
**NALM-6**	t(5;12) (q31q33;p12) ETV6-PDGFRB (2)
**RS4;11**	t(4;11) (q21;q23) MLL-AF4 (3)
**BV-173**	t(9;22) (q34;q11) BCR-ABL1(4)
**EU-3/697**	t(1;19)(q23;p13) E2A-PBX1 (5)
**REH**	t(12;21)(p13;q22) ETV6-AML1 (6)
**O18Z**	del(9)(p13) deletion CDKN2A/B (7)
**SUP-B15**	t(9;22) (q34;q11) BCR-ABL1 (8)
**TOM-1**	t(9;22) (q34;q11) BCR-ABL1 (8)
**NALM-20**	t(9;22) (q34;q11) BCR-ABL1 (8)
**KOPN-8**	t(11;19)(q23;p13.3) MLL-ENL (3)

Supplementary Table 2: Genotypes of B-ALL cell lines used in this study

Mouse BCR-ABL1-transformed *Foxo1*$^{fl/fl}$ pre-B cells were described earlier (9). The cells were cultured in Iscove’s modified Dulbecco’s medium (PAN Biotech, Aidenbach, Germany) supplemented with 10 % FBS, 2 mM L-glutamine, 100 U/mL penicillin, 100 µg/mL streptomycin 50 µM 2-mercaptoethanol at 37 °C and 7.5 % CO2. *Foxo1* knock-out was induced with 200nM 4-Hydroxy-tamoxifen (Merck KGaA, Darmstadt, Germany).

BCR-ABL1^{+} PDXs and murine OP9 stromal cells were kindly provided by Prof. Markus Müschen (Yale Medical School, USA) The refractory KRAS^{G12D} PDX JFK125R, the BCR-ABL1^{+} PDXs BLQ5 (derived from a relapsed patient after treatment with imatinib) and PDX2 (derived from a patient at point of diagnosis, additional *IKZF1* deletion) (10). PDX cells were cultured on OP9 cells in Alpha minimum essential medium (PAN

Biotech) containing 20 % FBS, 2 mM L-glutamine, 100 U/mL penicillin, 100 µg/mL streptomycin and 1 mM sodium pyruvate at 37 °C and 5 % CO_2.

Quantitative reverse-transcription PCR (qRT-PCR)

Isolation of RNA, cDNA synthesis and RT-qPCR were performed as described previously(11). For ensuring target specificity, primers were analyzed with Primer-BLAST (http://www.ncbi.nlm.nih.gov/tools/primer-blast/; RRID: SCR_003095) and subsequently synthesized by biomers.net (Ulm, Germany). Primer annealing temperature was set to 60 C and samples were measured in technical duplicates using the LightCycler 480 instrument (Roche, Basel, Switzerland). The relative expression of the target gene was calculated with the $2^{-\Delta\Delta CT}$ method. Human or mouse RPL13A was used as reference gene, respectively. ll primer sequences are listed in Supplementary Table 3.

Primer	Sequence
**human CCND1**	fwd: 5’- aagctgtgcatctacaccga -3’<br>rev: 5’- cttgagcttgttcaccagga -3’
**human CCND2**	fwd: 5’- gttagagtgcgcgaaggagt -3’<br>rev: 5’- gtggggcaaacagctagaga -3’
**human CCND3**	fwd: 5’- gtggccactaagcagaggag -3’<br>rev: 5’- cccttcaggcttagatgtgg -3’
**human c-MYC**	fwd: 5’- tcggattctctgctctcctc -3’<br>rev: 5’- tgttcctcctcagagtcgct -3’
**human RPL13A**	fwd: 5’- cggaccgtgcgaggtat-3’<br>rev: 5’- caccatccgctttttcttgtc -3’
**mouse Ccnd1**	fwd: 5’- cgaagtggagaccatccgcc -3’<br>rev: 5’- agggctccagggacaggaag -3’
**mouse Ccnd2**	fwd: 5’- ccgtacatgcgcaggatggt -3’<br>rev: 5’- gcagtcagcgggatggtctc -3’
**mouse Ccnd3**	fwd: 5’- ggctctccttccccctcaca -3’<br>rev: 5’- ttggagccccggacagaaga -3’
**mouse c-Myc**	fwd: 5’- tccctacccgctcaacgaca -3’<br>rev: 5’- cagcagcgagtccgaggaag -3’

**mouse Rpl13a**	fwd: 5’- cctgctgctctcaaggttgt -3’<br>rev: 5’- ggtacttccacccgacctc -3’

Supplementary Table 3: Primers used for qRT-PCR

Immunoblotting

Cell lines were lysed in SDS lysis buffer (62.5 mM Tris-HCl (pH 6.8), 2 % SDS, 10 % Glycerin, 50 mM DTT, 0.01 % bromphenol blue) and denatured for 5 minutes at 100 °C. Lysates were separated by SDS-PAGE and electrophoretically transferred to a 0.45 µm nitrocellulose membrane. The membrane was blocked for 15 minutes at 37 °C in 5 % non-fat dried milk/TBS-T (0.1 % Tween-20) and primary antibodies were incubated at 4 °C overnight. Next day, the membrane was washed twice in TBS-T (0.05 % Tween-20) and once in TBS (5 minutes per washing step). Horseradish peroxidase-conjugated secondary antibody was diluted 1:10000 (goat anti-rabbit, #31460, Thermo Fisher Scientific, Waltham, MA, USA; RRID: AB_228341) or 1:5000 (goat anti-mouse: 1:5000, #sc-2005, Santa Cruz Biotechnology, Dallas, TX, USA; RRID: AB_631736; donkey anti-goat: 1:5000, #sc-2020, Santa Cruz Biotechnology, Dallas, TX, USA; RRID: AB_631728) in 5 % non-fat dried milk/TBS-T (0.1 % Tween-20), and the membrane was incubated for 1h at RT in these solutions. Washing of the membrane was repeated as before and protein bands were visualized by addition of SuperSignal West Dura Extended Duration Substrate (Thermo Fisher) using the ChemiDoc imaging system (Bio-Rad, Hercules, USA). TUBB or ACTB protein expression levels were used as loading controls. Antibodies used are listed in Supplementary Table 4.

Target	Host species	Dilution	Manufacturer	Product ID
**ACTB-HRP**	Rabbit	1:2000	Santa Cruz	#sc-47778
**CCND1**	Rabbit	1:1000	Cell Signaling	29G2, #2978
**CCND2**	Rabbit	1:2000	Cell Signaling	D52F9, #3741
**CCND3**	Mouse	1:500	Cell Signaling	DCS22, #2936
**CDK8**	Rabbit	1:1000	Cell Signaling	P455, #4106
**FLAG M2**	Mouse	1:1000	Sigma-Aldrich	F1804
**FOXO1**	Rabbit	1:250	Cell Signaling	C29H4, #2880

**FOXO1-pS254**	Rabbit	1:1000	Cell Signaling	E1F7T, #84192
**LAMIN A/C**	Goat	1:1000	Santa Cruz	#sc-6215
**MYC**	Rabbit	1:500	Santa Cruz	#sc-788
**TUBB**	Rabbit	1:400000	Abcam	#ab6046

Supplementary Table 4: Antibodies used for immunoblot

Luciferase reporter assay

The promoter region of *CCND3* (NC_000006.12, https://www.ncbi.nlm.nih.gov, 22 May 2020) -1389 bp to +259 bp from the transcriptional start site (TSS) (long) or 0 bp to + 259 bp (short) from the transcriptional start site was gDNA isolated from RS4;11 cells into the pGL4.22 luciferase reporter vector into the XhoI restriction site using GIBSON assembly with the primers listed in Supplementary Table 5. The sequences of the cloned fragments were verified by Sanger sequencing (Eurofins Genomics, Ebersberg, Germany).

5x10^6 NALM-6 and RS4;11 cells were nucleofected with help of the AMAXA cell line kit T using the program C-005 on the Lonza Amaxa nucleofector IIb. An ubi-Renilla expression plasmid was co-transfected as an internal control. Immediately after transfection, cells were treated with 80nM of the FOXO inhibitor AS1842856. Luciferase activity was measured 24 h later using the Dual-Luciferase Reporter Assay System (Promega, Madison, Wisconsin, USA) and the Lumat LB 9507 tube luminometer (Berthold Technologies, Bad Wildbad, Germany). Firefly luciferase activity was normalized to the Renilla luciferase activity.

Long-Fwd	5’- ggtacctgagctcgctagccttacaggcgtgagctaccgc -3’
Long-Rev	5’- gaggccagatcttgatatccacccagcaccgatcccca -3’
Short-Fwd	5‘- ggtacctgagctcgctagccccgcgtggggcgggggct -3‘
Short-Rev	5’- gaggccagatcttgatatccagagccagtctccacccctgcagtggc -3‘

Supplementary Table 5: Primers used for cloning of the *CCND3* luciferase reporter

Electrophoretic mobility shift assay (EMSA)

First, a FOXO1 binding motif located in the *CCND3* (NC_000006.12, https://www.ncbi.nlm.nih.gov, 22 May 2020) promoter region was identified. In order to prove binding of FOXO1 to the *CCND3* promoter, two DNA-probes were designed,

both derived from -126 bp to -101 bp from the *CCND3* TSS. Both probes were 25 bp long and are listed in Supplementary Table 6. One probe (FOXO1-wt) contains the naturally occurring FOXO1 binding motif “GTAAACA” with the surrounding sequence taken from its position in the promoter region. The second one is flanked by the same sequences but contains a scrambled FOXO1 binding motif as described earlier (11). Probes were ordered biotinylated at the 5’ end and annealed by melting at 95 °C for 3 minutes, then annealed 70 °C for 10 minutes and slowly cooled down throughout the day. Nuclear extracts were harvested from HEK293T cells transfected with the PEI method(12) with either pFLAG-CMV2-Empty vector (EV) or pFLAG-CMV2-FhBox using the ThermoScientific (Waltham, USA) NE-PER™ Nuclear and Cytoplasmic Extraction kit 48h after transfection. The FhBox spans the highly conserved forkhead box domain containing the amino acids Q146 – A268 of human wild type FOXO3 (accession: NP_963853).

EMSA was performed using the ThermoScientific Light Shift kit, following the manufacturer’s instructions. Only binding buffer without any supplements was used for binding reaction. For super-shift, 2 µg anti-FLAG antibody (#F1804, Sigma-Aldrich, St. Louis, USA) PAGE was performed with a 5 % gel at 100V. Blotting was performed at 385mA for 1h. Chemiluminescence was detected using the Bio-Rad ChemiDoc MP device.

FOXO1-wt	fwd: 5’- ATCCAGGAAGTAAACAACATCATGA -3’<br>rev: 5’- TCATGATGTTGTTTACTTCCTGGAT -3’
FOXO1-mut	fwd: 5’- ATCCAGGAACGTGCAGACATCATGA -3’<br>rev: 5’- TCATGATGTCTGCACGTTCCTGGAT -3’

Supplementary Table 6: Oligonucleotide probes used for EMSA

ChIP (Chromatin immunoprecipitation)

ChIP was performed as we described previously (11). Briefly, NALM-6 cells were nucleofected with humanized biotin ligase BirA and either a construct expressing FOXO1 with an N-terminal biotinylation signal (bFOXO1) or empty vector (EV) control. The next day, chromatin was crosslinked with 37% formaldehyde, the reaction was quenched with 1 M glycin and cells were washed in 1X PBS. Extraction of nuclei was performed by douncing in Farnham lysis buffer. Chromatin was solubilized with RIPA

buffer and sonicated to an average fragment size of 500 bp. Pre-clear was performed with Protein G Dynabeads (Thermo Fisher, Waltham, USA) and precipitation of FOXO1-bound chromatin was done with streptavidin paramagnetic particles (Promega, Madison, Wisconsin, USA). The beads were washed first with 2 % SDS and then 500 mM LiCl wash buffer, DNA was extracted in the presence of 10 % Chelex (Bio-Rad, Hercules, CA, USA) in water. Fragments were purified using the QIAGEN PCR Purification kit (QIAGEN, Hilden, Germany). qRT-PCR analysis was performed with primers listed in Supplementary Table 7, using the QIAGEN Quantitect SYBR Green master mix. In order to quantify ChIP data, fold enrichment of the regions of interest (ROI) over the control Chromosome 12 (Ch12) region (R) was calculated. ChIP DNA was first normalized to input DNA by using the following formula: $R_i=2^{(ctInput-ctChIP)}$. Then, fold enrichment was calculated of *ROI* in samples transfected with bFOXO1 compared to EV: $R_{bFOXO1/EV} = R_{i(bFOXO1)}/R_{i(EV)}$. Next, R was calculated using the formula $R = R_{bFOXO1/EV\ ROI} / R_{bFOXO1/EV\ CH12}$.

CCND3	Fwd: 5’-CTGTCACAGTGCCTGACGTG-3’<br>Rev: 5’-GCAACTCCTCCACGTGCTA-3’
Ch12	Human Negative Control Primer Set 1<br>Active Motif (Carlsbad, CA, USA)

Supplementary Table 7: Primers used for qRT-PCR analysis of ChIP

Vectors and lentiviral transduction

shRNA constructs

shRNAs mined from the Broad Institute GPP Web Portal (https://portals.broadinstitute.org/gpp/public/) were cloned into the pRSI12-U6-sh-UbiC-TagRFP lentiviral vector (Cellecta, Mountain View, California, USA) as previously described(12). Scrambled control was described previously as well(12). ShRNA sequences are listed in Supplementary Table 8.

CCND3-shRNA1 (12)	Fwd: 5’-ACCGGCTAGGGTTATTGCATTTGGATGTTAATATTCATAGCATCCAAATGCAATAACCCTAGTTTTTTG -3’

	Rev: 5'- aattCAAAAAACTAGGGTTATTGCATTTGGATGCTATGAATATTAACATCCAAATGCAATAACCCTAGC -3'
CCND3-shRNA2 (13)	Fwd: 5'- ACCGGCAGACCAGCACTCCTACAGATGTTAATATTCATAGCATCTGTAGGAGTGCTGGTCTGTTTTTTG -3'
	Rev: 5'- AATTCAAAAAACAGACCAGCACTCCTACAGATGCTATGAATATTAACATCTGTAGGAGTGCTGGTCTGC-3'
Ccnd3-shRNA (mouse) cloneId=TRCN0000011978	Fwd: 5'- ACCGGGCCATGATGGTCAGAGAAATAGTTAATATTCATAGCTATTTCTCTGACCATCATGGCTTTTTTG -3'
	Rev: 5'- AATTCAAAAAAGCCATGATGGTCAGAGAAATAGCTATGAATATTAACTATTTCTCTGACCATCATGGCC -3'
CDK8-shRNA1 cloneId=TRCN0000199980	Fwd: 5'- ACCGGGTCCATGCACTGTTGCGAATGGTTAATATTCATAGCCATTCGCAACAGTGCATGGACTTTTTTG -3'
	Rev: 5'- AATTCAAAAAAGTCCATGCACTGTTGCGAATGGCTATGAATATTAACCATTCGCAACAGTGCATGGACC -3'
CDK8-shRNA3 cloneId=TRCN0000382350	Fwd: 5'- ACCGGATGGTGAAGTCACTATTATATGTTAATATTCATAGCATATAATAGTGACTTCACCATTTTTTTG-3'
	Rev: 5'- AATTCAAAAAAATGGTGAAGTCACTATTATATGCTATGAATATTAACATATAATAGTGACTTCACCATC -3'

Supplementary Table 8: Sequences of shRNAs used in this study

CRISPR/Cas9 editing

The sgRNA sequence targeting Exon 2 of *CCND3* was mined from the Broad Institute GPP Web Portal (https://portals.broadinstitute.org/gpp/public/) and cloned into lentiCRISPRv2 (a gift from Feng Zhang, Addgene plasmid #52961; http://n2t.net/addgene:52961 ; RRID:Addgene_52961) using the oligos listed in Supplemental Table 9.

gCCND3-fwd	5' caccgacacacgcacccgcaactgg -3'
gCCND3- rev	5'- aaacccagttgcgggtgcgtgtgtc -3'

Supplementary Table 9: Oligonucleotides used for cloning of a gRNA targeting *CCND3*

CCND3 over-expression.

We used SFFV-CCND3-GFP as described previously for ectopic expression of CCND3 (12).

Lentiviral transduction of human cell lines

HEK293T cells were transfected with the lentiviral plasmid of interest, the HIV-1 derived packaging plasmid p8.91 and the plasmid encoding the VSV-G envelope glycoproteins using polyethylenimine (PEI, Polysciences, Hirschberg a.d. Bergstraße, Germany). Transfection medium was swapped with fresh medium 8h later. Viral supernatant was harvested 48 h and 72 h after transfection. Cells were resuspended in viral supernatant and spinoculated at 2900 rpm for 2 h at 4 °C. Analysis was performed starting four days later.

Lentiviral transduction of murine cell lines

LentiX cells were transfected with the lentiviral plasmid of interest, p8.91 and VSV-G using $CaCl_2$-transfection (TaKaRa, Shiga, Japan). Medium was replenished the next day. Viral supernatants were harvested after 48 h and 72 h, pooled and concentrated by ultracentrifugation (25000 rpm, 90 min, 4 °C). Virus was diluted in IMDM and was loaded onto wells coated with 50 µg/ml retronectin (TaKaRa, Shiga, Japan) by centrifugation at 2000 x g for 120 min at 32 °C. Cells were then added to the wells and centrifuged at 300 x g for 30 min at 35 °C, incubated for two days at 37 °C, 7.5 % CO_2 before medium was replaced with fresh medium. Analyses started four days post transduction.

Lentiviral transduction of PDX cells

LentiX cells were transfected with the lentiviral plasmid of interest, p8.91 and VSV-G using $CaCl_2$-transfection (TaKaRa, Shiga, Japan). Medium was replenished the next day. Viral supernatants were harvested after 48h and 72h and pooled. Virus was loaded onto wells coated with 50 µg/ml Retronectin (TaKaRa, Shiga, Japan) by centrifugation at 2000xg for 120 min at 32 °C. Cells were then added to the wells and centrifuged at 300xg for 30 min at 35 °C, incubated for 24 h at 37 °C, 5 % CO_2 before medium was replaced with fresh medium. Analyses started four days post transduction.

Competitive growth assay

The competitive growth assay was performed as we described previously (12).

Briefly, cell lines were transduced with a shRNA expressing vector harboring the fluorescent marker RFP. The percentage of transduced cells was measured every 3

days, starting 4 days after transduction. The first measurement was set as 100 %, cell performance was measured relative to day 4 of the same sample. For analysis of growth performance of CCND3 over-expressing NALM-6 cells during treatment with palbociclib, cell number was analyzed simultaneously to flow cytometric analysis and calculated using the following formula, as described previously (12):

$$\frac{\mathrm{N} \times \%GFP^{+}\mathrm{cells}}{100}$$

N is the number of live cells per well and $\%GFP^+$ is the percentage of GFP^+ cells. Flow cytometric analysis was performed using the BD FACSCanto II (BD Biosciences, San Jose, CA, USA). Percentages of GFP^+ and RFP^+ cell populations were analyzed using the BD FACSDiva Software (RRID:SCR_001456) and Flow Jo (RRID: SCR_008520). For competitive growth assay experiments,

Apoptosis measurement

Four days after transduction with CCND3 shRNA or three days after treatment with palbociclib, cells were sorted with the S3e cell sorter (Bio-Rad, Hercules, USA) in order to enrich for 100 % transduced cells. Cells were then kept in culture for 2 days. Next, 1 x 10^6 cells were washed with Annexin V binding buffer (0.1 M Hepes, pH 7.4, 1.4 M NaCl, 25 mM $CaCl_2$), resuspendend in 76.5 µl staining solution (70 µl binding buffer, 5 µl Annexin V–FITC or Annexin V–APC (Immuno Tools, Friesoythe, Germany) and 1.5 µl PI (2 µg/mL, Sigma-Aldrich, St. Louis, MO, USA) and incubated for 15 min at room temperature in the dark. After addition of 500 µl binding buffer, samples were analyzed using the FACSCanto II flow cytometer (BD Biosciences). Apoptotic cells were defined as Annexin V or PI single or double positives, analyzed with FlowJo software (RRID: SCR_008520). Specific Apoptosis (SA) was calculated as 100 * (Exp - Con) / (100 - Con) as described previously (12).

Cell cycle analysis

Cells transduced with CCND3 shRNA were sorted 4 days after transduction with the S3e cell sorter (Bio-Rad, Hercules, USA). 1 x 10^6 cells were washed twice with PBS and fixed by adding 1 ml PBS and 3 ml ice-cold absolute ethanol dropwise to the sample while vortexing. After incubation on ice for 1 h, cells were centrifuged, resuspended in PBS containing 40 µg/mL PI (Sigma-Aldrich) and 100 µg/mL RNAseA

(Amersham Pharmacia, Piscataway, NJ, USA) and incubated for 30 min at 37 °C. Cell cycle analysis was performed by flow cytometry using the FACSCanto II (BD Biosciences) and FlowJo software (RRID: SCR_008520) as described previously (12).

IC50 calculation

Cell viability was calculated by MTT assay. $2x10^5$ cells were seeded per well in a 96 well plate in complete culture medium. Cells were treated with 2-fold serial dilutions of palbociclib with the highest concentration of 40 µM. Solvent control wells were incubated with dimethyl sulfoxide (DMSO), with volume corresponding to highest drug treatment. Positive control wells were treated with 8 µg/ml puromycin (#540222, Merck). Cells were incubated at standard conditions for 5 days. Then, 25 µl of the 5 mg/ml MTT solution (Thiazolyl Blue Tetrazolium Bromide, Sigma-Aldrich) was added and plates were incubated at 37 °C for 2 hours. Next, 100 µl lysis buffer per well (20 % SDS, 50 % dimethylformamide, 2 % acetic acid, 0.15 mM HCl, pH 4.7) was added and after an overnight incubation at standard conditions, the optical densities (OD) were measured at 570 nm wavelength using the SpectraMax 250 microplate reader (Molecular Devices, San Jose, CA, USA) and the SoftMax Pro 3.0 software (Molecular Devices; RRID: SCR_014240). Percentage of growth inhibition at a given drug concentration was calculated as $(1 - OD_{drug} - OD_{puromycin}) / OD_{DMSO} * 100$. The IC_{50} was calculated using GraphPad Prism software (RRID: SCR_002798).

RNA-sequencing

RNA was isolated from $1x10^6$ cells either 4 days after transduction with scrambled or shCCND3 or 3 days after treatment with 1 µM palbociclib or equivalent amount of DMSO, using the RNeasy kit (Qiagen, Hilden, Germany). Integrity of RNA was checked with Agilent 2100 bioanalyzer (Agilent, Santa Clara, USA) and quality was further controlled with Nanodrop (ThermoFisher, Waltham, USA). cDNA library construction and sequencing were performed by the Beijing Genomics Institute (BGI, Shenzhen, China) with BGISEQ-500. Data filtering was performed with SOAPnuke software (version 1.5.2), mapping of reads to the reference genome hg38 was done with Bowtie2 (version 2.2.5). Differentially expressed genes were calculated and discovered with RSEM (version v1 2.12) and DEseq2. Additional analysis was

performed with Qlucore Omics Explorer (version 3.6). Parameters for differential genes analysis were $p<0.05$, $q<0.1$ and fold change > 1.5. Three biological replicates per cell line were analyzed.

Additional statistical methods

Estimation of the sample size was done according to the method described by Blainey et al. (14).

Supplementary references

1. Hruz T, Laule O, Szabo G, Wessendorp F, Bleuler S, Oertle L, et al. Genevestigator V3: A Reference Expression Database for the Meta-Analysis of Transcriptomes. Adv Bioinformatics [Internet]. 2008 Jul 8 [cited 2021 Apr 22];2008:1–5. Available from: https://pubmed.ncbi.nlm.nih.gov/19956698/
2. Wlodarska I, Aventín A, Inglés-Esteve J, Falzetti D, Criel A, Cassiman JJ, et al. A new subtype of pre-B acute lymphoblastic leukemia with t(5;12)(q31q33;p12), molecularly and cytogenetically distinct from t(5;12) in chronic myelomonocytic leukemia. Blood [Internet]. 1997 Mar 1 [cited 2021 Apr 22];89(5):1716–22. Available from: http://ashpublications.org/blood/article-pdf/89/5/1716/1411220/1716.pdf
3. Stong RC, Korsmeyer SJ, Parkin JL, Arthur DC, Kersey JH. Human acute leukemia cell line with the t(4;11) chromosomal rearrangement exhibits B lineage and monocytic characteristics. Blood [Internet]. 1985 Jan 1 [cited 2021 Apr 22];65(1):21–31. Available from: http://ashpublications.org/blood/article-pdf/65/1/21/1639230/21.pdf
4. Pegoraro L, Matera L, Ritz J. Establishment of a Ph1-positive human cell line (BV173). J Natl Cancer Inst [Internet]. 1983 [cited 2021 Apr 22];70(3):447–53. Available from: https://pubmed.ncbi.nlm.nih.gov/6572735/
5. Drexler HG, Macleod RA. History of leukemia-lymphoma cell lines. Hum Cell. 2010;23(3):75–82.
6. Matsuo Y, Drexler HG. Establishment and characterization of human B cell precursor-leukemia cell lines. Leuk Res. 1998 Jul 1;22(7):567–79.
7. Eckhoff EM, Queudeville M, Debatin K-M, Meyer LH. A Novel B Cell Precursor ALL Cell Line (018Z) with Prominent Neurotropism and Isolated CNS Leukemia in a NOD/SCID/huALL Xenotransplantation Model. Blood. 2009 Nov 20;114(22):1630–1630.
8. Drexler HG, MacLeod RAF, Uphoff CC. Leukemia cell lines: In vitro models for the study of Philadelphia chromosome-positive leukemia. Vol. 23, Leukemia Research. Elsevier Ltd; 1999. p. 207–15.
9. Abdelrasoul H, Vadakumchery A, Werner M, Lenk L, Khadour A, Young M, et al. Synergism between IL7R and CXCR4 drives BCR-ABL induced

transformation in Philadelphia chromosome-positive acute lymphoblastic leukemia. Nat Commun [Internet]. 2020;11(1):3194. Available from: https://doi.org/10.1038/s41467-020-16927-w

10. Chan LN, Murakami MA, Robinson ME, Caeser R, Sadras T, Lee J, et al. Signalling input from divergent pathways subverts B cell transformation. Nature [Internet]. 2020;583(7818):845–51. Available from: https://doi.org/10.1038/s41586-020-2513-4

11. Vogel MJ, Xie L, Guan H, Tooze RM, Maier T, Kostezka U, et al. FOXO1 repression contributes to block of plasma cell differentiation in classical Hodgkin lymphoma. Blood [Internet]. 2014 Nov 13 [cited 2021 Apr 30];124(20):3118–29. Available from: http://ashpublications.org/blood/article-pdf/124/20/3118/1381549/3118.pdf

12. Wang F, Demir S, Gehringer F, Osswald CD, Seyfried F, Enzenmüller S, et al. Tight regulation of FOXO1 is essential for maintenance of B-cell precursor acute lymphoblastic leukemia. Blood. 2018 Jun;131(26):2929–42.

13. Radulovich N, Pham N-A, Strumpf D, Leung L, Xie W, Jurisica I, et al. Differential roles of cyclin D1 and D3 in pancreatic ductal adenocarcinoma. Mol Cancer [Internet]. 2010;9(1):24. Available from: https://doi.org/10.1186/1476-4598-9-24

14. Blainey P, Krzywinski M, Altman N. Replication. Nat Methods [Internet]. 2014;11(9):879–80. Available from: https://doi.org/10.1038/nmeth.3091

2. Supplementary Figures

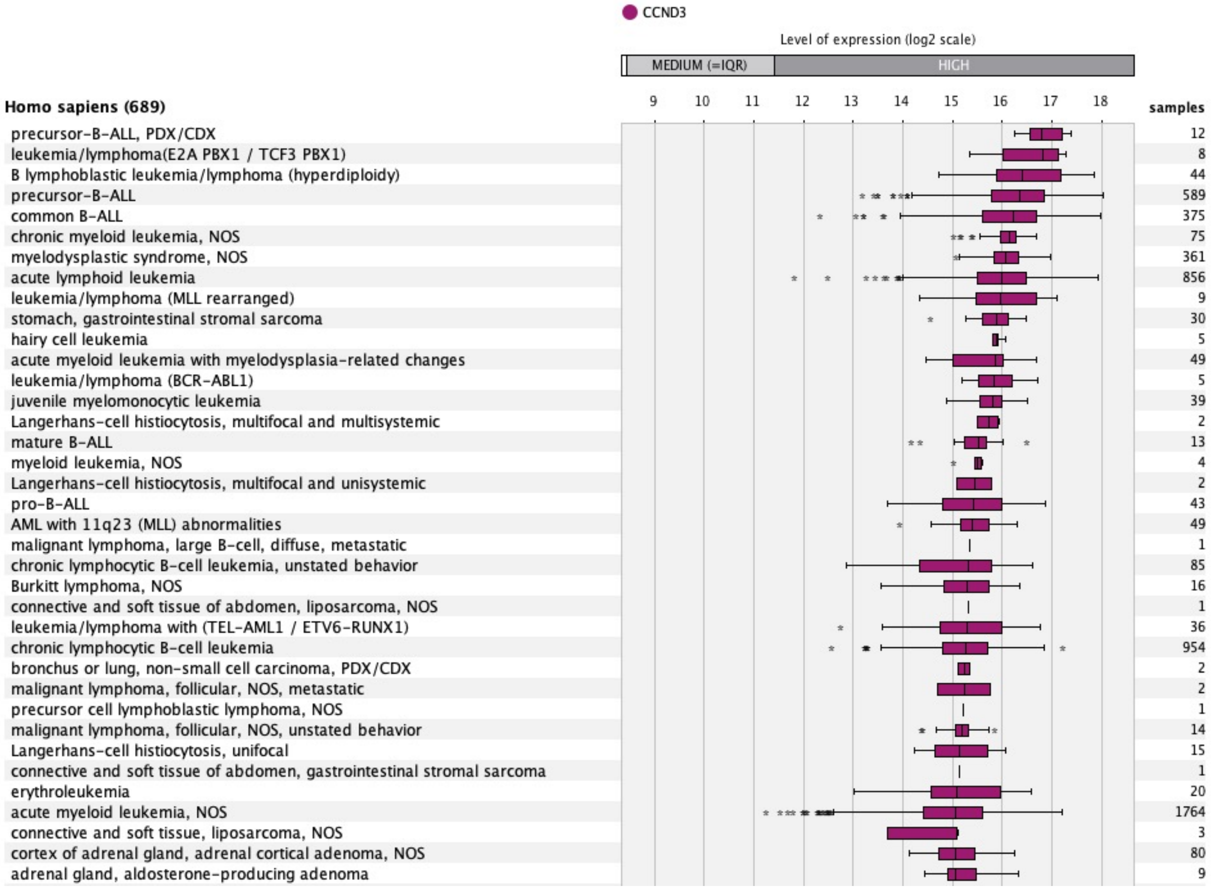

Supplementary Fig. 1. CCND3 mRNA expression of patient samples

Data mining from the publicly available database GENEVESTIGATOR shows CCND3 mRNA is expressed at the highest levels in various types of B-ALL. Shown are the top 37 CCND3 expressing types of cancer out of 689 indexed.

A

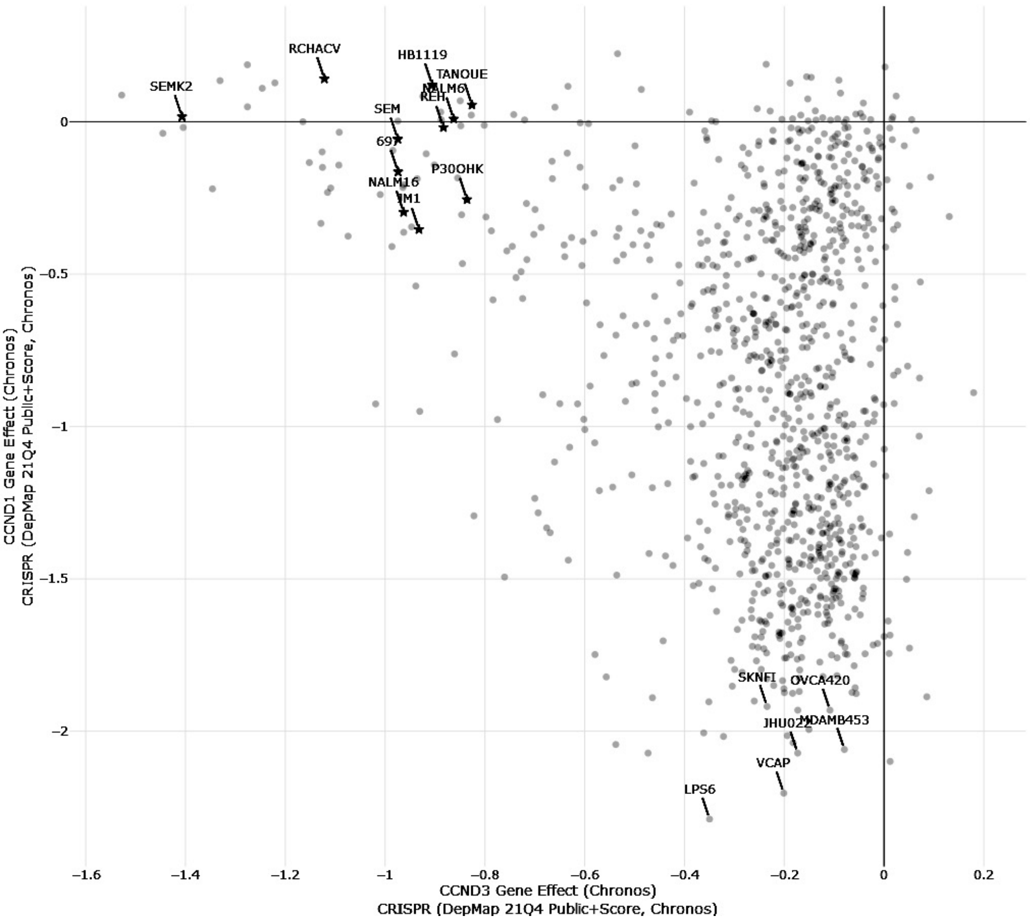
CCND1 Gene Effect (Chronos)
CRISPR (DepMap 21Q4 Public+Score, Chronos)
CCND3 Gene Effect (Chronos)
CRISPR (DepMap 21Q4 Public+Score, Chronos)
0
−0.5
−1
−1.5
−2
−1.6
−1.4
−1.2
−1
−0.8
−0.6
−0.4
−0.2
0
0.2
RCHACV
HB1119
TANOUE
SEMK2
NALM6
REH
SEM
697
P30OHK
NALM16
JM1
SKNFI
OVCA420
JHU022
MDAMB453
VCAP
LPS6

B

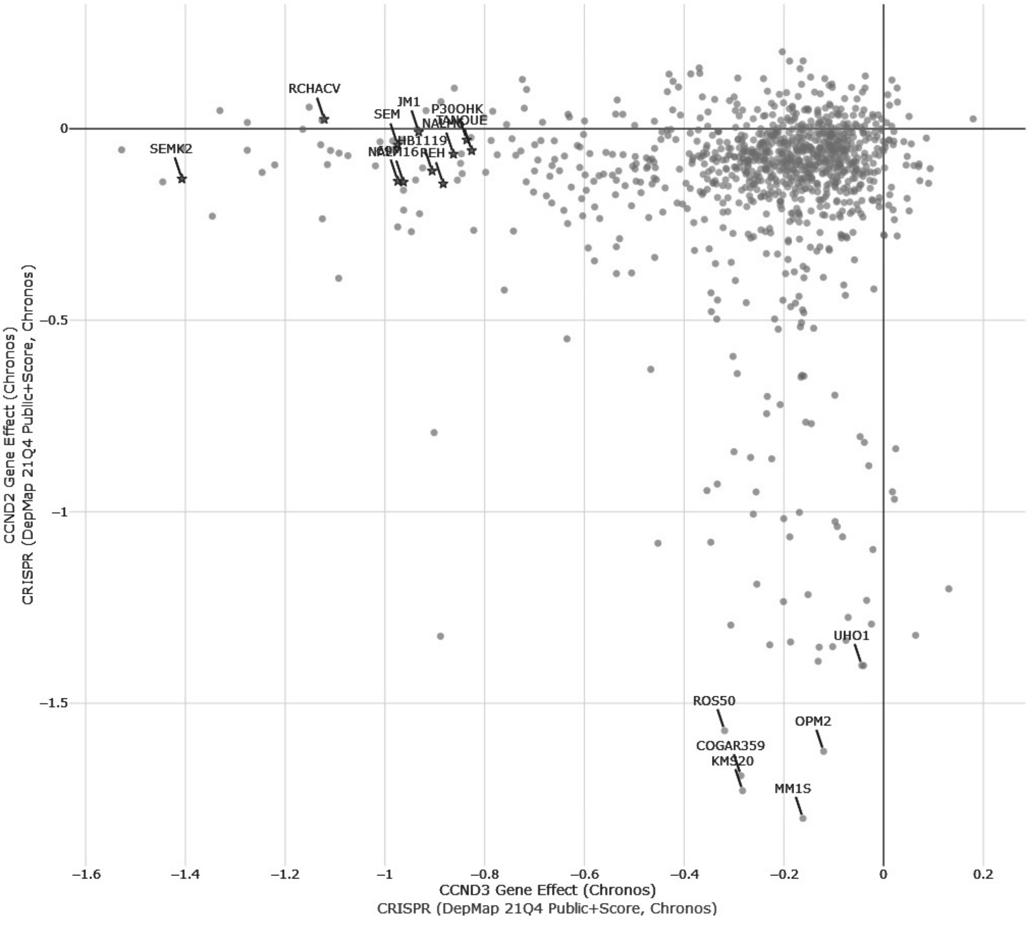
CCND2 Gene Effect (Chronos)
CRISPR (DepMap 21Q4 Public+Score, Chronos)
CCND3 Gene Effect (Chronos)
CRISPR (DepMap 21Q4 Public+Score, Chronos)
0
−0.5
−1
−1.5
−1.6
−1.4
−1.2
−1
−0.8
−0.6
−0.4
−0.2
0
0.2
RCHACV
JM1
P30OHK
SEM
TANOUE
SEMK2
HB1119
NALM16
REH
UHO1
ROS50
OPM2
COGAR359
KMS20
MM1S

Supplementary Fig. 2. Strong dependency of B-ALL on CCND3.

Genetic dependency data based on publicly available CRISPR/Cas9 knockout screen data were downloaded from Broad DepMap portal database (https://depmap.org/portal/download/; Dataset DepMap 21Q3 Public+Score, Chronos). The data are available for B-ALL cell lines SEM, SEMK2 (KMT2A-AFF1), 697 and RCH-ACV (TCF3-PBX1), REH (ETV6-RUNX1), NALM-6 (ETV6-PDGFRB; BCR-ABL-like), JM-1, HB1119, and P300HK (pre-BCR^{+} cell lines, which do not harbor common oncogenic translocations). Negative scores correspond to stronger dependency. A score of 0 is indicates that gene that is not essential whereas the median of common essential genes is equal to -1.

(A) CRISPR/Cas9-knockout screens show that B-ALL cell lines are dependent on CCND3 but not on CCND1. Cell lines most dependent on CCND1 are comprised of the solid tumor cell lines LPS6 (liposarcoma), VCAP (prostate), JHU022 (head and neck squamous cell carcinoma), MDAMB453 (mammary carcinoma), OVCA420 (ovary carcinoma) and SKNFI (skin cancer). (B) CRISPR/Cas9 knockout screens show that B-ALL cell lines are dependent on CCND3 but not CCND2. Cell lines most dependent on CCND2 include multiple myeloma (MM1S, KMS20, OPM2), COGAR359 (teratoid/rhabdoid tumor), ROS50 (DLBCL) and UHO1 (classical Hodgkin lymphoma).

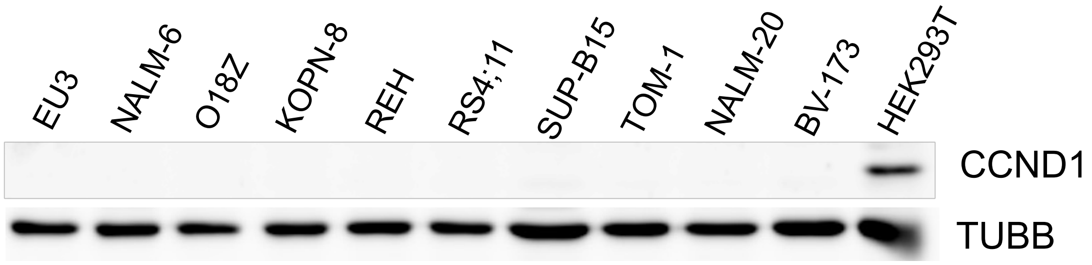

Supplementary Fig. 3. Protein expression levels of CCND1 in different B-ALL cell lines

Immunoblot was performed for B-ALL cell lines with various underlying driver mutations. Image is representative of n=2.

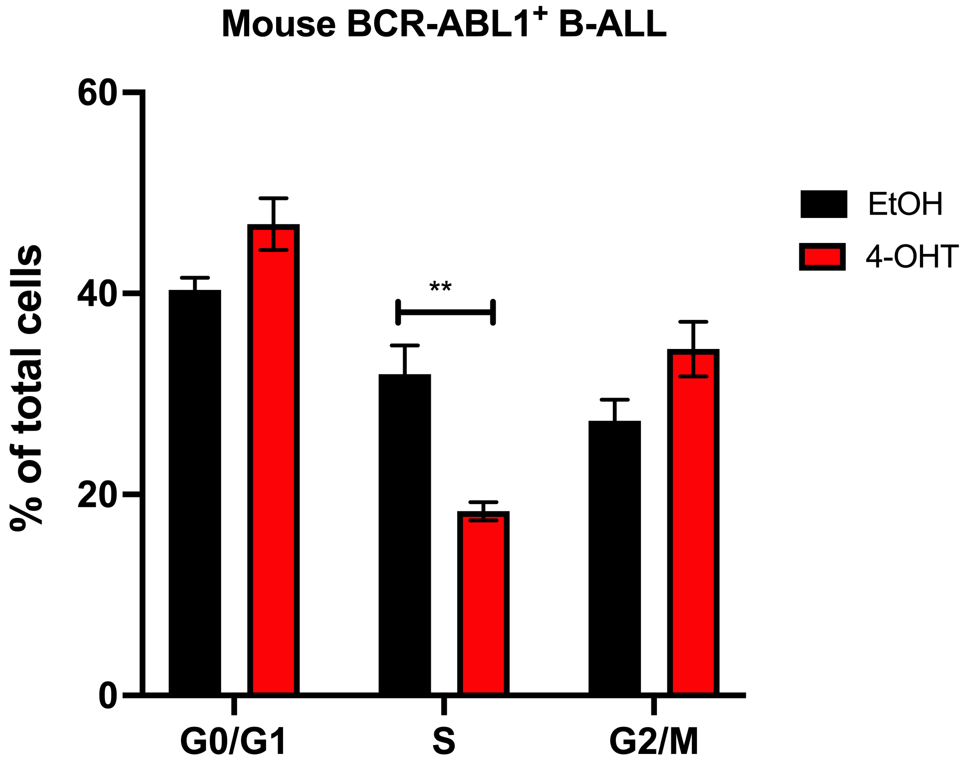

Supplementary Fig. 4. Foxo1 deletion induces cell cycle arrest in murine Foxo1$^{fl/fl}$ Cre^{ERt2} BCR-ABL1^{+} B-ALL cells

Deletion of *Foxo1* in BCR-ABL1-transformed murine B-ALL cells after induction with 200 nM 4-OHT stops cell cycle progress at G0/G1 leading to a decrease of cells in S phase. Data shown as mean ± SD, n=3. **=p<0.01

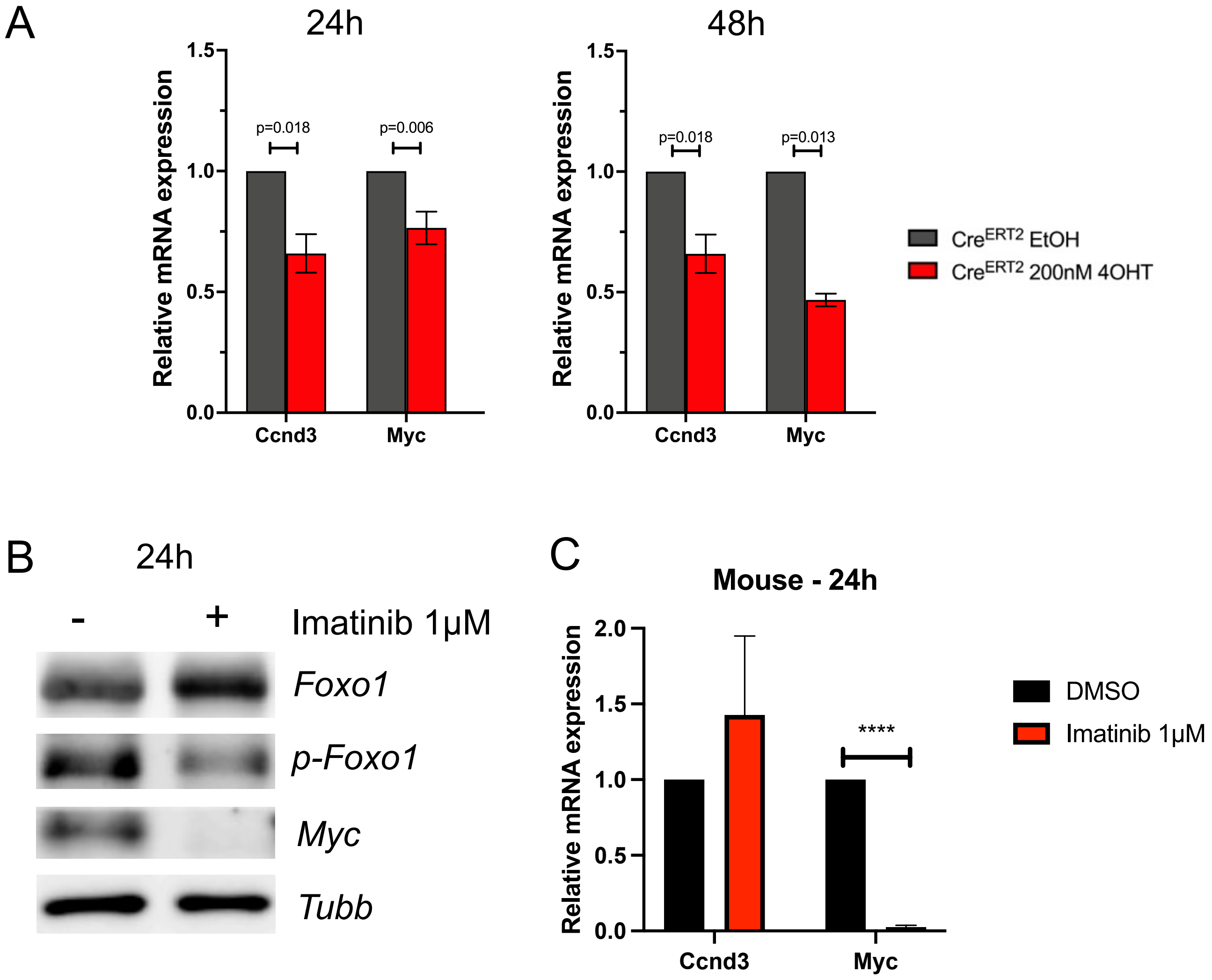

Supplementary Fig. 5. Ccnd3 transcription is independent of Myc

(A) Inducible knock-out of Foxo1 in BCR-ABL1-transformed murine *Foxo1*$^{fl/fl}$ Cre^{ERT2} pre-B cells decreases both *Ccnd3* and *Myc* mRNA levels. Cells were treated with 200 nM 4-OHT to induce *Foxo1* deletion and harvested 24 h and 48 h later. Data shown as mean ± SD, n=3. (C) Treatment of BCR-ABL1-transformed murine pre-B cells with imatinib increases Foxo1 levels and efficiently abolishes Myc protein expression. Image representative of n=3. (D) *Ccnd3* mRNA expression remains stable while *Myc* expression is significantly reduced. Data shown as mean ± SD, n=3.

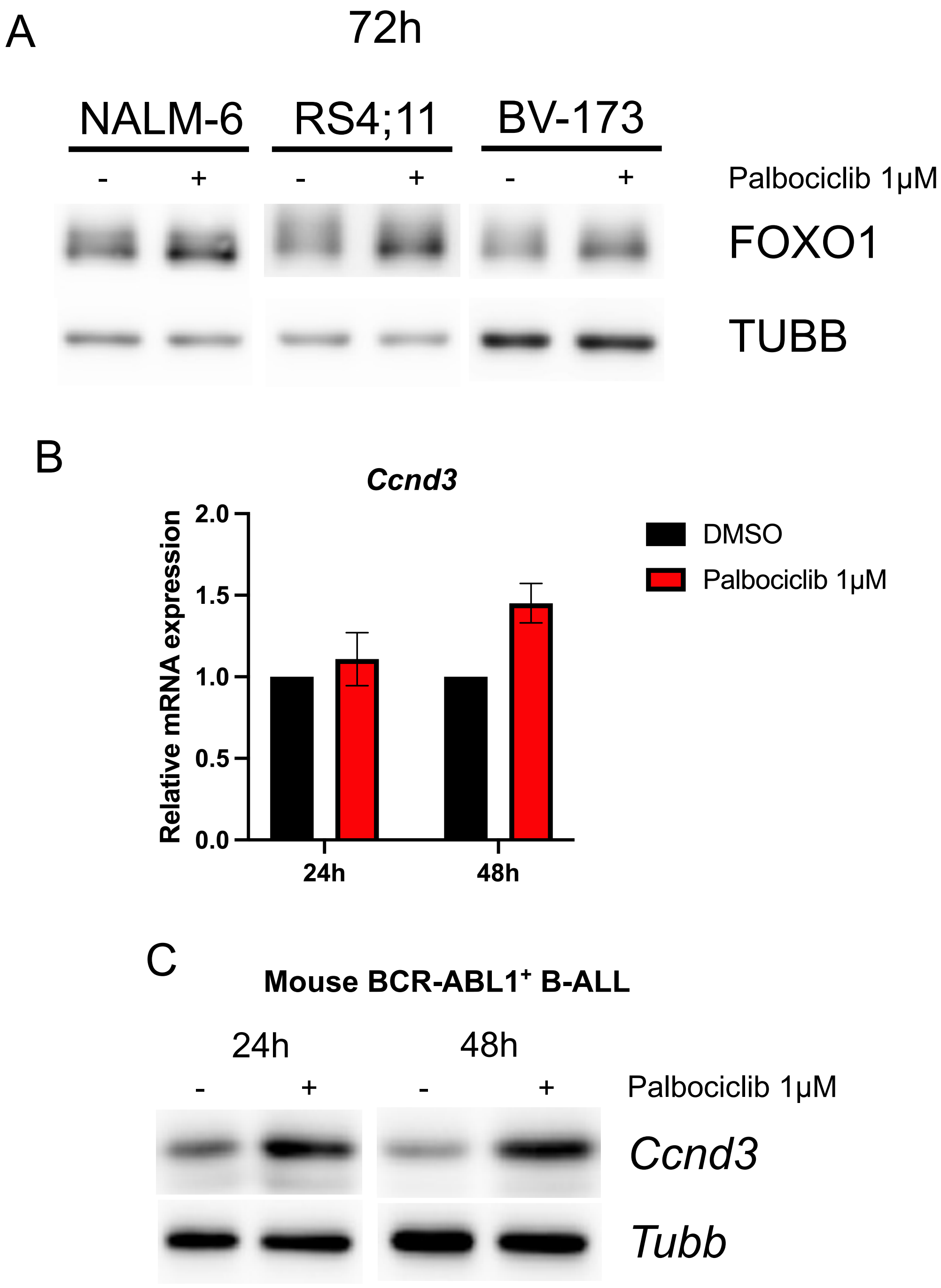

Supplementary Fig. 6. Palbociclib does not repress expression of Ccnd3

(A) B-ALL cell lines were treated with 1μM palbociclib for 72h increases FOXO1 protein levels. Image is representative of n=3.

(B) Treatment of BCR-ABL1-transformed murine B-ALL cells with palbociclib increases Ccnd3 transcription. Data shown as mean ± SD, n=3.

(C) Ccnd3 protein expression increases after 24h and 48h of treatment with palbociclib in BCR-ABL1-transformed murine B-ALL cells. Image is representative of n=3.

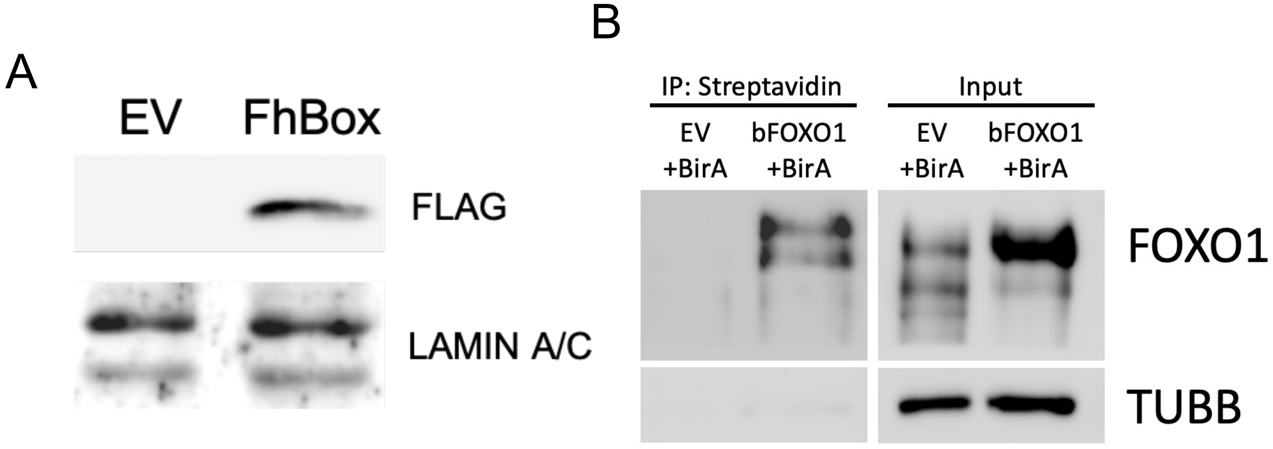

Supplementary Fig. 7. Transfection controls for EMSA and ChIP
(A) Nuclear extracts of HEK293T cells transfected with either pFLAG-CMV2-Empty vector (EV) or pFLAG-CMV2-FhBox (FhBox) were analyzed by immunoblot to prove efficiency of transfection. LAMIN A/C was used as loading control Image is representative of n=2.
(B) NALM-6 cells were transfected with human BirA and a pcDNA3.1+ vector expressing either N-terminally biotinylation-signal modified FOXO1 (bFOXO1) or with empty vector (EV). Cells were lysed the next day after transfection and biotinylated FOXO1 was captured with streptavidin-coated magnetic beads. FOXO1 and TUBBN were detected by imunoblot. Input samples were taken before the streptavidin pulldown. Image is representative of n = 2.

chr6:42,046,365-42,050,252

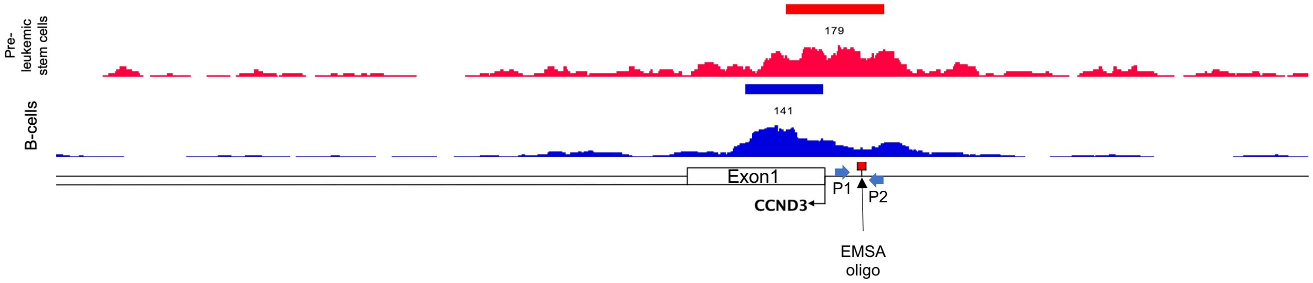

Supplementary Fig. 8. ChIP-sequencing data of FOXO1
Publicly available ChIP-sequencing data were mined from https://chip-atlas.org/. Data were analyzed using Integrated Genome Viewer (IGV 2.1.3) and Genepalette (v.2.1.1). Displayed are the BIGWIG tracks of FOXO1-chromatin binding in pre-leukemic stem cells (Red tracks, GEO accession: GSM2136846) and B-lymphocytes (Blue tracks, GEO accession: GSM1668935). Peak calls displayed are significant at $q < 0.05$, number above peaks indicates call length in bp. The FOXO binding motif (GTAAACA) that was included in the oligo probe used for EMSA, located -126 bp from the TSS, is indicated. Primers used for ChIP (Fig. 2F) are indicated with blue arrows as P1 and P2, flanking the FOXO binding motif in the CCND3 promoter.

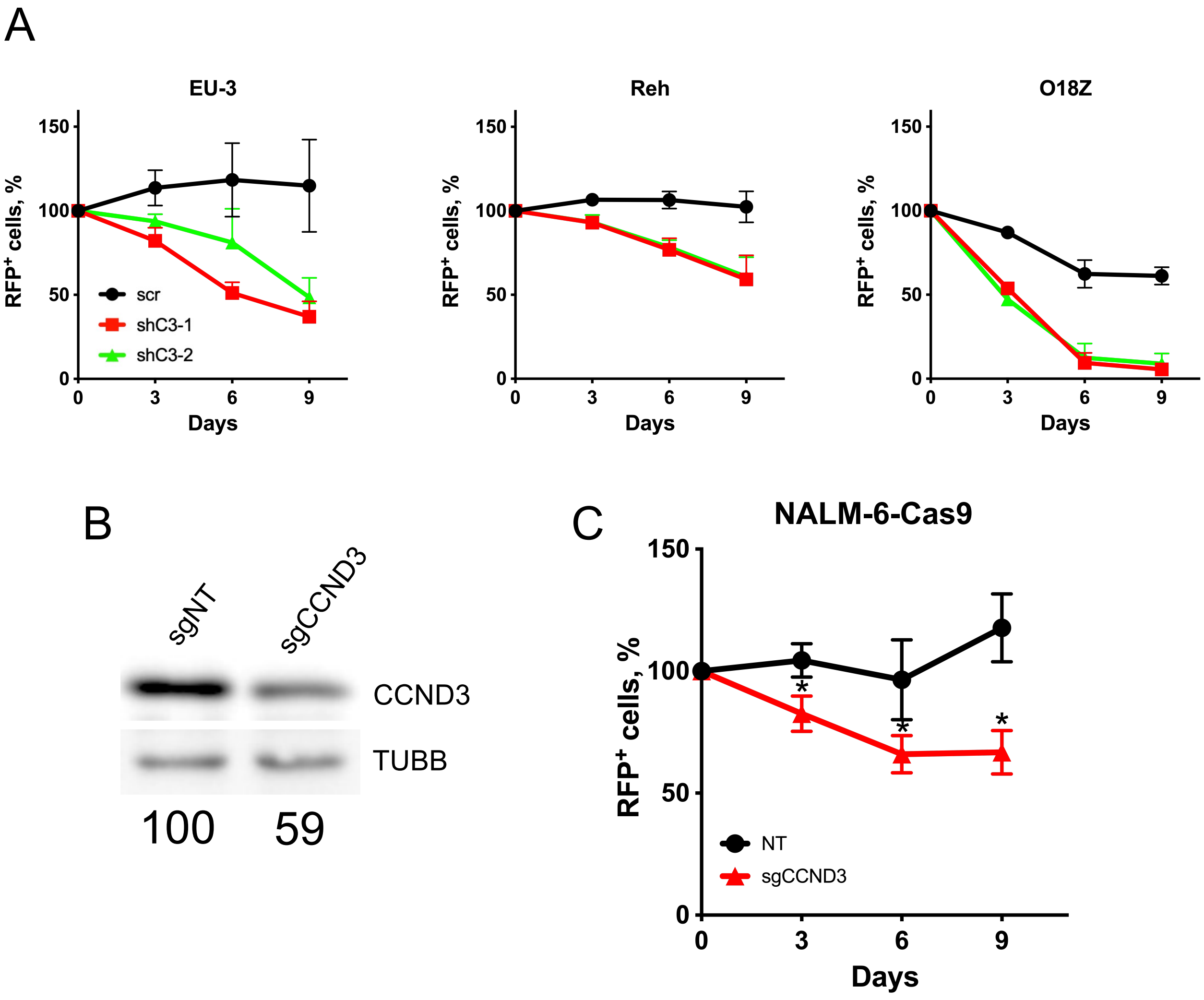

Supplementary Fig. 9. CCND3 shRNA knockdown and CRISPR/Cas9 knockout in B-ALL cell lines

(A) B-ALL cell lines were transduced with either shRNAs targeting *CCND3* (shC3-1/-2) or with a non-targeting control shRNAs, co-expressing fluorescent marker RFP. Cellular growth was tracked by flow cytometry. All B-ALL cell lines show growth disadvantage after *CCND3* knockdown. Data shown as mean ± SD, n=3. (B) CRISPR/Cas9 knockout of CCND3 in NALM-6 cells verified by immunoblot, knockout efficiency approx. 40%. Protein expression level is indicated below, relative to sgNT. Image representative of n=3. (C) CRISPR/Cas9 knockout of *CCND3* induces growth inhibition in NALM-6 cells. Data shown as mean ± SD, n=3. *=$p<0.05$

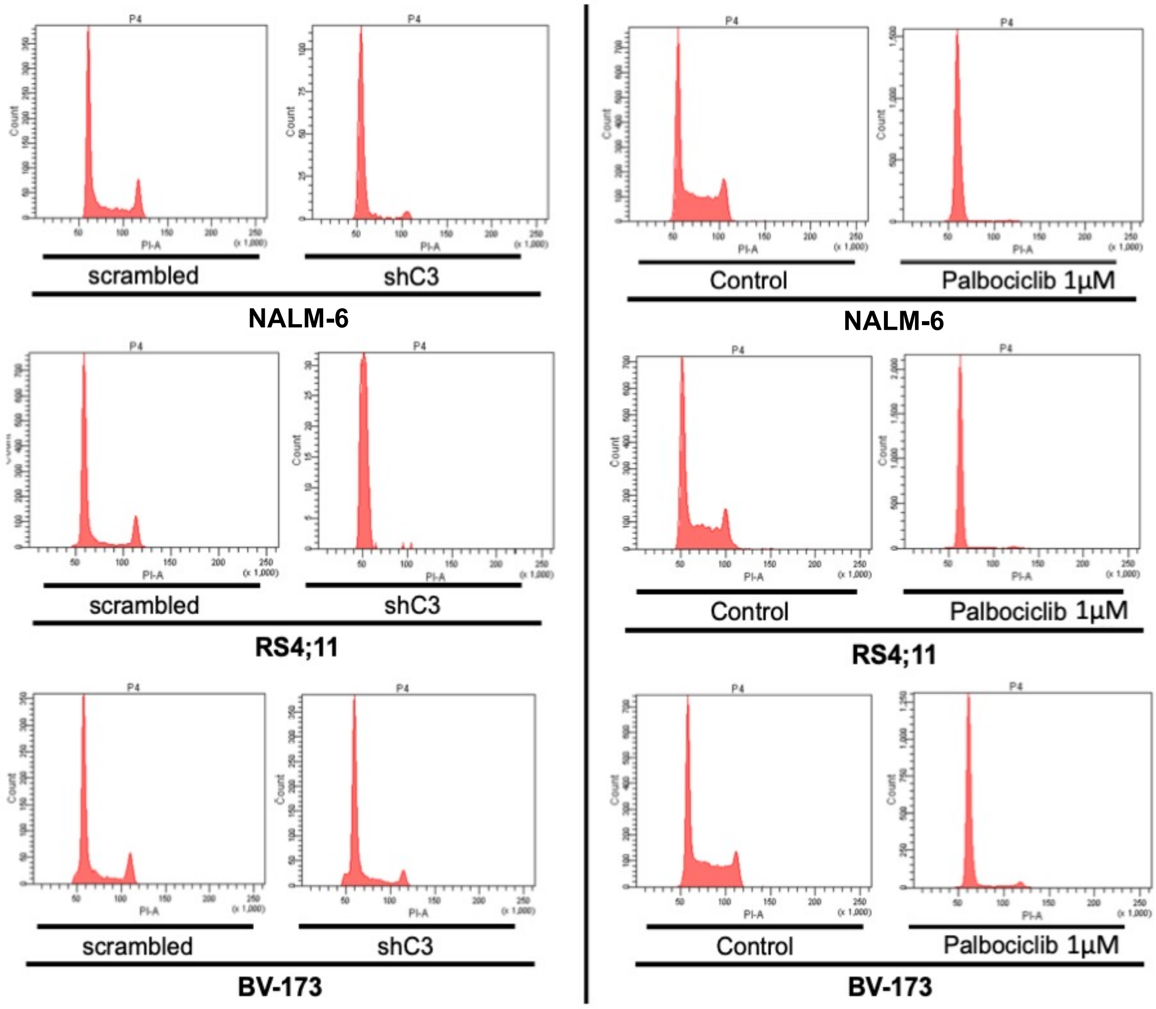

Supplementary Fig. 10. CDK4/6 inhibition and CCND3 knockdown induce cell cycle arrest

NALM-6, RS4;11 and BV-173 B-ALL cell lines were either treated with 1μM palbociclib for 72h or lentivirally transduced with CCND3 targeting shRNA, sorted after four days and fixed and stained with propidium iodide after three more days. DNA content of the cells was analyzed by flow cytometry. G0/G1 cell cycle arrest was observed for both treatments. Image representative of n=3.

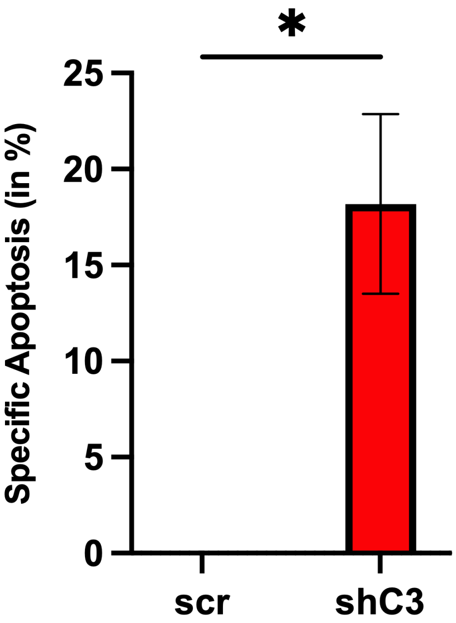

Supplementary Fig. 11. *Ccnd3* shRNA knockdown induces apoptosis in murine BCR-ABL1-transformed pre-B cells.
murine BCR-ABL1-transformed pre-B cells display significant induction of apoptosis after shRNA knockdown of *Ccnd3*. Data shown as mean ± SD, n=3. *=p<0.05

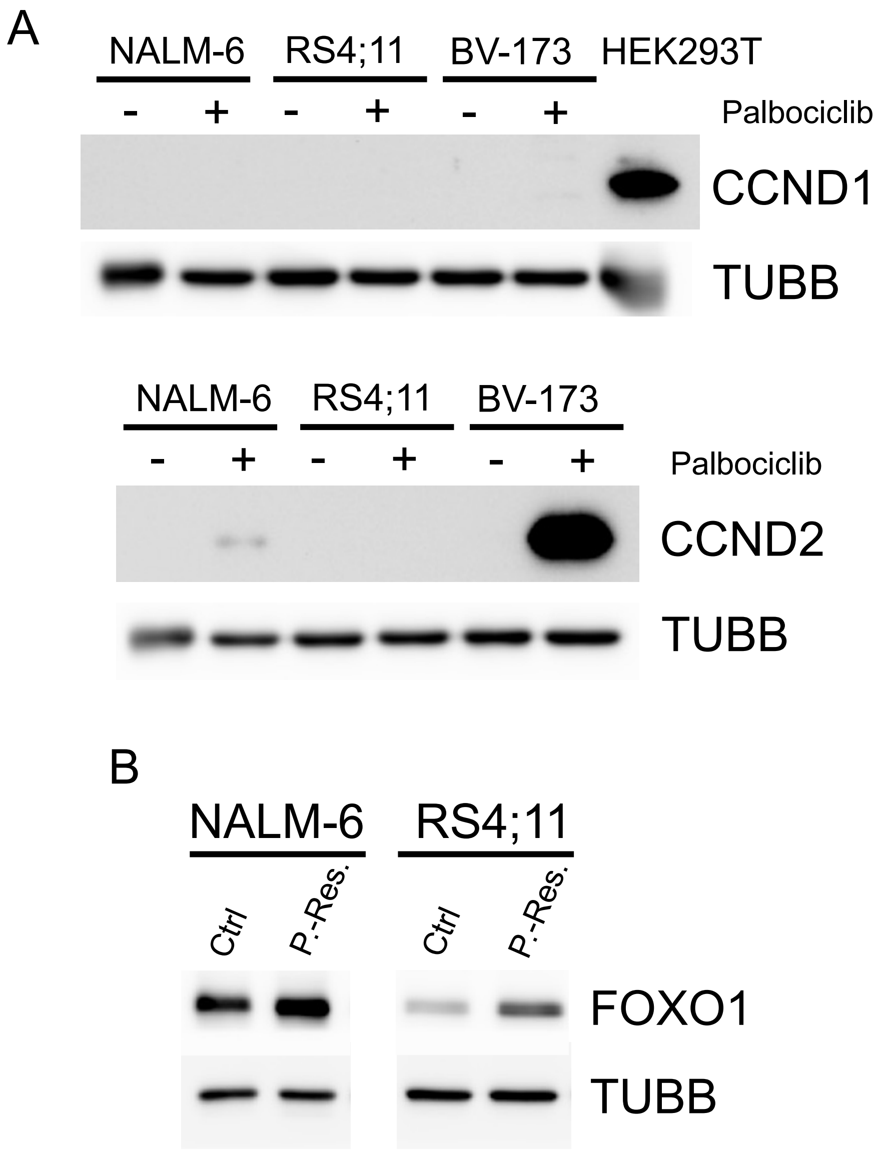

Supplementary Fig. 12. Alterations of CCND1, CCND2, and FOXO1 expression in B-ALL cell lines induced by acute and prolonged treatment with palbociclib

(A) NALM-6, RS4;11 and BV-173 were treated with 1 µM palbociclib for 72h. CCND1 is not detectable before or after treatment, HEK293T protein lysates were loaded as a positive control for CCND1. CCND2 is strongly upregulated in BV-173, slightly in NALM-6 and not detectable in RS4;11. Image is representative of n=2.

(B) NALM-6, RS4;11 resistant to palbociclib show upregulation of FOXO1 protein. Image representative of n=2.

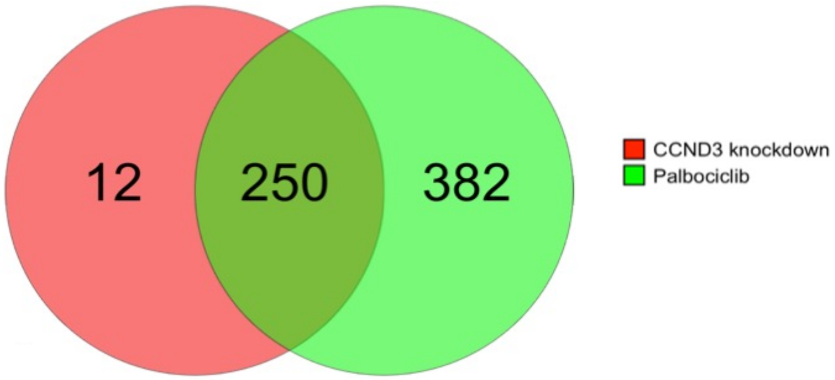

Supplementary Fig. 13. Comparison of differentially expressed genes after Palbociclib or shCCND3

Differentially expressed genes in NALM-6, RS4;11 and BV-173 after either 3 days treatment with 1µM Palbociclib or 4 days after transduction with CCND3 targeting shRNA were compared with a Venn approach. 12 genes were exclusively regulated after CCND3 knockdown. Differentially expressed genes were identified using Qlucore Omics Explorer (v3.6) with $p < 0.01$ and $q < 0.1$.

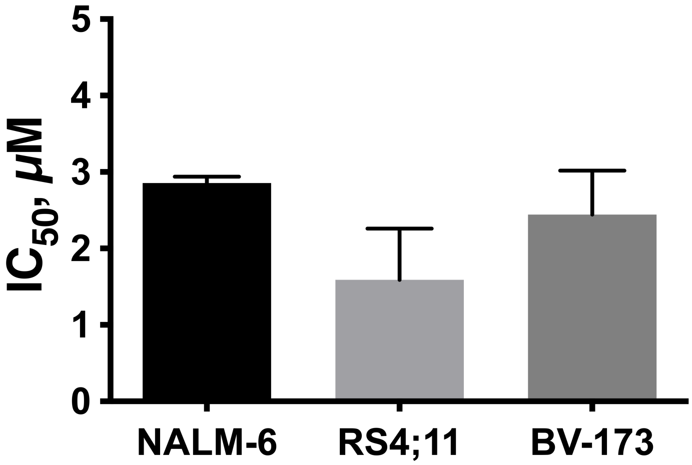

Supplementary Fig. 14. B-ALL cell lines are insensitive to CDK8 kinase inhibitor SEL120

(A) IC_{50} was determined by MTT assay for NALM-6 (ETV6-PDGFRB), RS4;11 (MLL-AF4) and BV-173 (BCR-ABL1) cell lines. Data shown as mean ± SD, n=2.
